# Altered stress and fear responses in the VPA rat model of autism: Behavioral dissociation across tactile, nociceptive, and social contexts

**DOI:** 10.1371/journal.pone.0353839

**Published:** 2026-07-17

**Authors:** Debora Hashiguchi, Ana Luiza Dias, Rodrigo Neves Romcy-Pereira

**Affiliations:** 1 Laboratory of Neural Circuit Plasticity, Brain Institute, Federal University of Rio Grande do Norte, UFRN, Natal, Rio Grande do Norte, Brazil; 2 Laboratory of Computational Neuroscience, Brain Institute, Federal University of Rio Grande do Norte, UFRN, Natal, Rio Grande do Norte, Brazil; Dr. Anjali Chatterji Regional Research Institute for Homeopathy, INDIA

## Abstract

Altered sensitivity to environmental stimuli is a core feature of autism spectrum disorder (ASD) that may drive vulnerability to stress-related and anxiety disorders. To investigate this relationship, we evaluated autonomic and behavioral responses to tactile, nociceptive, and social stressors in juvenile Wistar rats prenatally exposed to valproic acid (VPA), a well-established model of ASD. VPA-exposed and saline-treated control (CTL) rats were subjected to a behavioral paradigm measuring defecation, freezing, and ultrasonic vocalizations (USVs) in response to handling, electro-tactile stimulation, classical fear conditioning, and an emotional contagion task. Compared to CTLs, VPA rats exhibited sustained hyperdefecation during handling and a higher prevalence of defecation during electro-tactile stimulation, independent of freezing changes. During fear conditioning, VPA rats demonstrated a delayed onset but subsequent enhancement of freezing responses, alongside disrupted temporal coordination between freezing and defecation behaviors. 22-kHz USVs positively correlated with freezing across both groups. In the emotional contagion task, observing a distressed conspecific increased freezing prevalence and suppressed vocalization rates in both cohorts; however, these effects were prolonged in VPA rats, which showed persistent freezing and an earlier reduction in vocalization rate. These findings indicate that VPA-treated rats display heightened stress reactivity, habituation deficit and suggest disrupted coordination of fear responses, supporting the VPA model as a relevant tool for investigating the neurobiological basis of stress vulnerability and social dysfunction in ASD.

## Introduction

The ability to respond to environmental changes is a fundamental characteristic of human existence. Excessive worry, fear, and heightened sensitivity to threats characterize stress and anxiety disorders. Evidence indicates that individuals with ASD are especially susceptible to experiencing anxiety [1–7]. Studies suggest that innocuous stimuli are more frequently appraised as stressful by affected individuals compared to normotypical individuals, which may result in behaviors similar to the characteristics inherent to ASD, such as difficulties in coping with changes, abnormal responsiveness to sensory stimuli and social demands, thus increasing the vulnerability of these individuals to stress disorders [1,8–12].

In humans and animals, the primary arousal system in the human body is the autonomic nervous system (ANS). The ANS responds rapidly to stress by activating and inhibiting its sympathetic and parasympathetic branches. These responses manifest in a variety of physiological changes, including increased heart rate, blood pressure, respiratory rate, pupil dilation, and colonic motility [13–17]. These responses collectively prepare the body to confront or avoid the stressor, commonly known as the Fight-or-flight response. Behavioral responses to these physiological changes include increased alertness and focus, enhanced sensory perception, and suppression of nonessential functions. Consequently, studies use changes in cardiac activity, sweating, skin temperature, and colonic motility as indicators to assess ANS functionality [18–22]. Increased fecal pellet release, driven by increased colonic motility, and freezing behavior, which involves the integration of central nervous system, ANS, and sensorimotor functions, have been observed in stressed rats [23–26]. However, the behavioral response elicited varies depending on the level of disturbance in ANS homeostasis. Furthermore, stress-induced emission of ultrasonic vocalizations (USVs) within the frequency range of 20–35 kHz, also known as 22-kHz calls, has been widely correlated with the level of stress experienced by the animal [27,28]. Calls lasting less than 100 ms are typically associated with acute distress, while longer calls, which can extend up to 3000 ms, are frequently observed under sustained stressful conditions [29–33]. All of these behaviors are recognized as stress signals and considered vital for social communication, particularly in signaling danger within a group.

The valproic acid (VPA) rat model is extensively employed as a preclinical model of autism spectrum disorder (ASD) due to its recapitulation of key neural and behavioral alterations characteristic of the condition [34–37]. Despite its widespread application, the model’s validity for investigating stress susceptibility remains underexplored. This study aimed to assess the feasibility of employing the VPA rat model for translational research on stress responses to tactile, nociceptive, and adverse social stimuli. To achieve this, four experimental paradigms were employed: (a) a manipulation test and (b) an electro-tactile sensitivity test to evaluate stress responses to tactile stimuli; (c) a fear conditioning paradigm to assess stress responses to nociceptive stimuli; and (d) an emotional contagion paradigm to investigate stress responses to negative social stimuli.

## Materials and methods

### Animals

Experimental animals were obtained by breeding male and female Wistar rats from the Brain Institute Animal Facility (BISIC) at the Federal University of Rio Grande do Norte (UFRN). Breeding pairs were >60 days old and weighed between 250–300 g at the time of mating. On gestation day 12.5, 14 pregnant females were injected with either sodium valproate (n = 8; 250 mg/mL saline solution, 500 mg/Kg, i.p., VPA; Sigma-Aldrich P-4543) or saline (n = 6; 0.15M NaCl) solutions. A group of pregnant females (n = 9) was left undisturbed for the generation of naive animals to be used in the emotional contagion paradigm (as demonstrators). In total, we used 23 litters generated by mating 17 females with 14 males. Six females were mated twice and contributed with two litters (first litter CTL and second litter VPA, n = 1; first litter CTL and second litter naive, n = 1; first litter naive and second litter VPA, n = 3; or first litter VPA and second litter naive, n = 1). After birth, pups were left in their homecage with the dam until weaning at postnatal day 21 (P21).

Newborn rats used in the tactile sensitivity tests were transferred at P21 to new clean cages (3–6 littermates/cage) without sexual segregation. A total of 58 animals were included in these experiments: 28 VPA-treated rats (15 females and 13 males) and 30 CTL rats (15 females and 15 males). Behavioral tests were conducted between P33 and P37. Newborn animals used in the nociceptive and negative social experiments were transferred at P21 to new clean cages in pairs. In each cage, a VPA-treated or CTL rat was housed with a naive rat of the same age and sex. A total of 122 animals were included in these experiments: 31 VPA-treated rats (15 females and 16 males), 30 CTL rats (15 females and 15 males) and 61 naive rats (30 females and 31 males). The same cohorts of VPA and CTL animals were used for the nociceptive and negative social experiments. Behavioral tests were performed on P38 and P42. Offspring from the same litter was balanced distributed across the groups and mixed-effects analyses were performed to explicitly account for litter-level clustering (S1 Table).

All VPA-treated rats showed a characteristic tail kink due to the VPA embryological effect. All experimental procedures used in this study were approved by the Ethics Committee on the Use of Animals (CEUA-UFRN, protocol nº 017/2020). Throughout the experiments, all animals were kept in polypropylene boxes (55 x 46 x 30 cm) in a conditioned environment, with temperature set to 24°C, 70% humidity, a 12h/12h light-dark cycle (lights-on: 7am; lights-off: 7 pm), with food and water freely available.

### Behavioral tests

#### Touch sensitivity test.

To investigate touch sensitivity, we used gentle handling as a stimulus. Over a period of four days, rats aged P33 to P36 were transferred from the vivarium to an experimental room within their housing boxes. Once in the experimental room, each rat was individually removed from its cage and handled by the experimenter. Handling involved four sessions/day of one minute each, during which the animals received gentle hand strokes on their fur while held in the experimenter’s hand. Between sessions, animals were transferred back to their home cage where they stayed for five minutes before the next session. Handling sessions were carried out at the end of the lights-on phase, between 4 pm and 7 pm. Once the handling was completed, the animals were returned to the vivarium.

#### Electro-tactile sensitivity test.

To investigate electro-tactile sensitivity of the glabrous skin of the paws, we used footshocks as stimuli. The experimental apparatus consisted of a shock chamber measuring 32 cm in length, 25 cm in width, and 30 cm in height, featuring a metallic grid floor for the delivery of the electrical foot-shock stimuli. The chamber had a translucent roof with small openings on the sides, two metallic walls, and two translucent walls, through which video recordings were obtained. Video monitoring captured the entire area of the apparatus, through two high-definition webcams (Logitech C920). Foot-shock stimuli were applied through the grid by a programmable digital stimulus generator (STG 4002 Multi Channel Systems) controlled by software (MC stimulus 4002). Each stimulus consisted of a rectangular pulse of 1 sec duration and varying amplitude (50–550 μA; 50 μA steps). A digital ultrasonic microphone (Ultramic 250K, Dodotronics) was also positioned through the opening at the roof of the chamber to record vocalizations. Microphone and video camera recordings were synchronized to the foot-shocks by an Arduino device programmed to activate a light flash and a sound buzz simultaneously to the electrical stimulus.

All animals were tested one day after the last handling session, which was used as habituation to the experimenter and to the experimental room. The experiment took place at the end of the lights-on phase, between 4 pm and 7 pm and was conducted under dim light. For the experiment, the rats were placed in the middle of the apparatus facing one of the metal walls. After a period of 90 sec of free exploration, single electrical stimuli of 1 sec were applied with increasing intensity in 50 µA steps with random inter-stimulus intervals between 30 and 90 sec. The test was considered completed when the animal displayed jump or flee, for two consecutive stimuli.

#### Nociceptive stress response.

To investigate the nociceptive stress response, we used a contextual fear conditioning paradigm with repeated unpredictable footshocks. The apparatus consisted of an operant conditioning chamber (32L x 25W x 30H cm each) with a metal grid floor, a translucent roof with small openings on the sides, surrounded by two metallic and two translucent walls. Electrical foot shock stimuli were applied by a programmable digital stimulus generator (STG 4002 MultiChannel Systems) controlled by software (MC stimulus 4002). Video monitoring captured the entire area of the apparatus, through a high-definition webcam (Logitech C920) positioned above the apparatus. A digital ultrasonic microphone (Ultramicrophone 250K, Dodotronic; 16 bits ADC; 250kHz sampling rate) was positioned through the opening at the roof of the chamber to record vocalizations at approximately 30 cm from the chamber floor. Microphone and video camera recordings were synchronized to the foot-shocks by an Arduino device programmed to activate a light flash and a sound buzz simultaneously to the electrical stimulus. Videos recordings in .MP4 format and audio recordings in.WAV format were stored on a hard disk for posterior analysis.

The experiment took place at the end of the lights-on phase, between 2 pm and 5 pm and was carried out under dim light. At P38, rats were placed in the middle of the apparatus facing one of the metal walls. Following a 4-min period of free exploration that served as baseline (B0), five electrical shock stimuli of 1-sec duration and random inter-stimulus interval 0–180 sec were applied. The experiment finished 4 minutes after the last shock was applied. We used a shock intensity (750 μA) that corresponded to two times the nociceptive threshold defined during the electro-tactile sensitivity test. At the end of the experiment, each rat was transferred to a new clean (neutral) cage for one hour before returning to its home cage.

#### Social stress response.

In order to investigate the animal’s response to a cagemate stress demonstration, we used the emotional contagion paradigm, in which an experimental animal (observer, OBS) observing a conspecific (demonstrator, DEM) undergoes a series of aversive foot shocks. The apparatus used consisted of two compartments: a conditioning chamber (shock chamber) adjacent to a neutral chamber (32L x 25W x 30H cm) was separated by a translucent plexiglass wall with two rows (4 and 6 cm above the floor) of 8 open roles each (1 cm diameter). An ultrasonic microphone was positioned in the shock chamber through a roof aperture in the chamber (30 cm from the floor) to record vocalizations. Control software and equipment synchronization were the same as previously described. Audio (.WAV files) and video recordings (.MP4 files) were saved and stored in hard disk for posterior analysis of freezing and vocal behaviors. Habituation and testing were conducted during the lights-off phase, between 7:00 p.m. and 3:00 a.m., under dim light.

Over the following two days of fear conditioning, OBS animals were habituated to the neutral chamber for 10 minutes each day (P39-P40). They were placed in the center of the neutral box, facing the shock chamber, and allowed to freely explore the apparatus and then, returned to their home cages. At P41, both OBS and DEM were brought to the experimental apparatus. The DEM animal was placed in the center of the shock chamber, facing one of the side walls, while the OBS was placed in the center of the neutral chamber, facing one of the side walls. Following a 4-min period of free exploration, which served as a B0, five electrical shock stimuli of 1-sec duration, 750 µA intensity, delivered at random inter-stimulus interval 0–360 sec, were applied to the DEM animal. In order to maximize the OBS-to-DEM attention, all shock stimuli were delivered to the DEM only when the OBS animal faced the DEM. Following the last stimulus, both animals remained for an additional 4 min in the apparatus. Throughout the experiment, the OBS animal was able to make visual contact with the DEM through a clear plexiglass division. All experiments were conducted under dim light conditions.

In order to assign the identity of the call emitter while using a microphone in the DEM chamber, we defined a power intensity threshold based on vocal recordings under two experimental paradigms conducted in a separate group of animals. Vocalizations above −76 dB were considered as originating from animals in the same chamber where the microphone was placed (DEM chamber). Calls with power below −85 dB were considered predominantly originating from animals in the adjacent chamber (OBS chamber). In summary, high intensity vocalizations were considered DEM calls, whereas low intensity vocalizations were considered OBS calls. Vocalizations with power between −76 dB (lower cut-off to demonstrator calls) and −85 dB (higher cut-off to observer calls) were removed from our analyses, in order to minimize chamber assignment ambiguity, as they could have originated either from the same chamber of the microphone or from the adjacent chamber. Calls in this range comprised 9.8% of all vocalizations (2086/21388 calls) produced during the emotional contagion experiment (8.4% of CTL calls and 11.2% of VPA calls), therefore sparing 91.2% of calls to our analysis (S1 Fig).

### Behavioral analysis

Quantification of fecal pellets was recorded at the end of each handling session and immediately after all other experiments. To maintain objective quantification of variables, video and audio files (.MP4 and.WAV) were randomly coded and analyzed by a skilled experimenter.

For the determination of the tactile threshold, the following behaviors were considered: grid investigation and paw retraction. The tactile threshold was defined as the lowest current at which the animal exhibited any of the previous behaviors. For the determination of the nociceptive threshold, the following behaviors were considered: jumping and fleeing. The nociceptive threshold was defined as the lowest current at which the animal displayed any of these behaviors. A freezing episode was identified as the behavioral state where the animal remained completely still without any intentional movement for a duration exceeding 3 sec. It was quantified as the relative freezing time throughout the experiment. The analyzes of nociceptive and social negative experiments compared measurements during the B0 with either their combination during all shock blocks (B1-5) or individually with their values per block (B1 to B5). Following B0, each subsequent block (B1-B4) extended from 2s prior to a shock to 2s before the next shock. The last block (B5) extended from 2s before the fifth shock to 4 min after this shock. All video recordings were blindly analyzed by behavioral logging using BORIS software v.7.13.8.

The vocal behavior during the experiments was analyzed by quantifying the number of USVs produced and their acoustic properties. The analysis consisted of four steps: (1) Call detection, (2) Curation, (3) Segmentation and (4) Feature extraction. They were performed using software routines developed in our laboratory and a custom-modified version of DeepSqueak software v2.6.2. Automatic USV detection was performed by computing a time-dependent entropy score of the power distribution across frequencies in the audio spectrogram with time resolution = 2 ms; frequency resolution = 65 Hz; frequency range, 20–100 kHz using the Chronux software package (Bokil et al., 2010). USV curation and segmentation consisted in a software-assisted refinement of USV time and frequency boundary boxes, which were performed by a trained investigator. The software performed a pre-identification of the USVs, which could be confirmed or rejected by the investigator. The investigator could include USVs that were not identified by the software or exclude regions of noise incorrectly identified as USVs. The criteria used to consider a putative USV were as follows: minimum frequency of 20 kHz, maximum frequency of 125 kHz, minimum duration of 5 ms, and a maximum interval between consecutive signals of 30 ms. We quantified the number of calls produced per animal as well as, call duration and fundamental frequency bioacoustic properties of each vocalization. During the fear conditioning experiment, we focused on vocalizations of < 35-kHz and duration > 200 ms.

In the behavioral tests, animals were considered “responsive” based on minimal engagement criteria specific to each paradigm. When evaluating freezing behavior, an animal was considered responsive if it exhibited at least one freezing episode (≥ 3 s) during the experimental session. When evaluating ultrasonic vocalizations, responsiveness was defined as the emission of at least one ultrasonic call. Correlation analyses involving freezing behavior included all animals exhibiting at least one freezing episode, irrespective of ultrasonic vocalization emission or fecal pellet release.

### Statistics

We applied parametric tests when data distributions conformed to normality criteria, otherwise non-parametric tests were used. For proportions and percentages, data were z-transformed before applying a parametric test. Correlation between freezing behavior, fecal pellet release, and ultrasonic vocalization (USV) emission were analyzed using linear mixed-effects models with group and sex as fixed effects and litter as a random effect. Spearman rank correlations were additionally used to assess pairwise associations between variables. To evaluate potential litter effects in the social stress response experiment, we applied linear mixed-effects models including litter as a random effect and additionally performed a sensitivity analysis in which outcomes were averaged within each litter and statistical comparisons were repeated using litter means as independent observations. Correction for multiple tests was applied to original p-values when necessary, by computing a new set of adjusted p-values through the two-stage False Discovery Rate (FDR) proposed by Benjamini, Krieger and Yekutieli [38]. Statistical significance level was set at 0.05. Statistical analyses were conducted using GraphPad Prism software (v9.0.0) and Matlab.

## Results

### Increased sensitivity and delayed habituation to repeated handling in VPA-treated rats

**Fig 1:**
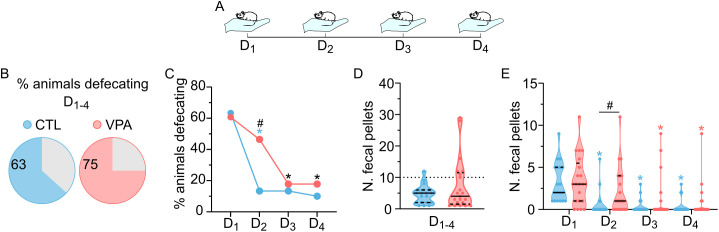
Stress responses elicited by handling. **(A)** Schematic illustration of the experimental design. Animals underwent four 1-min handling sessions separated by 5-min intervals, once daily for four consecutive days. **(B)** Percentage of animals releasing fecal pellets across all experimental days (*p* > 0.050). **(C)** Percentage of animals releasing fecal pellets on each experimental day (^#^*p* = 0.025; CTL: *p < 0.001; VPA: *p = 0.007). **(D)** Total number of fecal pellets released across all experimental days (*p* > 0.050). The dotted line indicates the reference value of 10 fecal pellets. **(E)** Number of fecal pellets released on each experimental day (^#^*p* = 0.025; CTL: *p ≤ 0.003; VPA: *p = 0.007). Panels B and C: Fisher’s exact test. Panel D: Mann–Whitney test. Panel E: Mann–Whitney and Friedman tests. Panels B and C: nCTL = 30 (15 females, 15 males), nVPA = 28 (15 females, 13 males). Panel D and E: nCTL = 19 (9 females, 10 males); nVPA = 21 (10 females, 11 males). ^#^ indicates significant between-group differences; * indicates significant within-group differences compared with Day 1 (D1).

Across four handling sessions (Fig 1A), CTL and VPA-treated rats showed a similar overall prevalence of defecation behavior, defined as the release of at least one fecal pellet (Fig 1B). However, day-by-day analysis revealed a greater proportion of VPA-treated rats releasing fecal pellets on the second experimental day relative to CTL rats (Fig 1C). CTL rats exhibited a rapid reduction in defecation prevalence after the first session, indicating early habituation to handling stress. In contrast, VPA-treated rats showed a delayed reduction that became evident only from the third day onward.

To further assess stress-response magnitude, we quantified the number of fecal pellets released by responsive animals. No overall group differences were detected across the four experimental days (Fig 1D). Nevertheless, 33% of VPA-treated rats released more than 10 fecal pellets, compared with only 6% of CTL rats, suggesting greater stress susceptibility in the VPA group. Consistent with this interpretation, VPA-treated rats released more fecal pellets than CTL rats on the second experimental day (Fig 1E). Temporal analyses additionally showed that CTL rats reduced pellet release from the second day onward, whereas VPA-treated rats displayed delayed habituation beginning only on day 3.  No sex-related differences were observed in either the prevalence or magnitude of the defecation response (S2 Fig).

### Increased susceptibility of VPA-treated rats to electro-tactile stimulation

**Fig 2:**
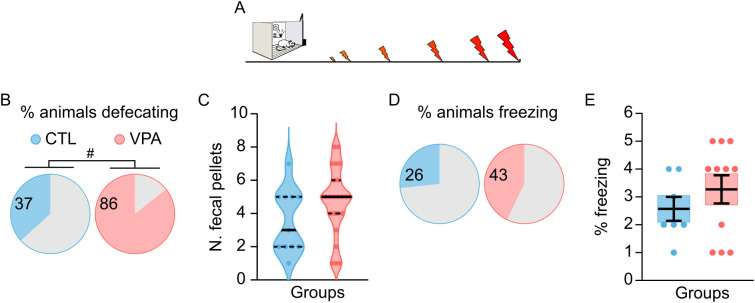
Stress responses elicited by electro-tactile stimuli. **(A)** Schematic illustration of the experimental design. Rats were placed in the center of the apparatus facing one of the metal walls. After 90 s of free exploration, 1-s electrical stimuli were delivered at progressively increasing intensities in 50-µA increments, with random interstimulus intervals ranging from 30 to 90 s. The test was terminated after the occurrence of nociceptive responses to two consecutive stimuli. **(B)** Percentage of animals releasing fecal pellets (^#^*p* = 0.001). **(C)** Number of fecal pellets released (*p* > 0.050). **(D)** Percentage of animals displaying freezing behavior (*p* > 0.050). **(E)** Percentage of time spent freezing (*p* > 0.050). Panels B and D: Fisher’s exact test. Panel C: Mann–Whitney test. Panel E: Student’s *t*-test. Panels B and D: nCTL = 30 (15 females, 15 males), nVPA = 28 (15 females, 13 males). Panel C: nCTL = 11 (7 females, 4 males), nVPA = 24 (14 females, 10 males).; Panel E: nCTL = 8 (5 females, 3 males), nVPA = 12 (6 females, 6 males). ^#^ indica*t*es significant between-group differences.

During the electro-tactile sensitivity test (Fig 2A), a larger proportion of VPA-treated rats released fecal pellets compared with CTL rats (Fig 2B), indicating increased stress susceptibility. However, the number of pellets released per responsive animal did not differ between groups (Fig 2C). Similarly, freezing behavior showed no significant between-group differences, either in the proportion of animals exhibiting freezing or in freezing duration among responsive animals (Fig 2D and 2E).

Additionally, assessments of tactile and nociceptive thresholds indicated no significant differences between VPA-treated and CTL animals (S3A Fig). In order to assess sex differences, we compared the behavior of females and males in each group. Our results showed no sex differences in stress susceptibility, magnitude of defecation response or freezing behavior in the VPA or CTL group. Similarly, no sex differences were detected in the nociceptive threshold. However, female CTL rats exhibited higher tactile thresholds compared to their male counterparts, whereas no sex differences were observed among VPA-treated animals (S3B Fig).

### Delayed onset and enhanced freezing responses in VPA-treated rats during fear conditioning

**Fig 3:**
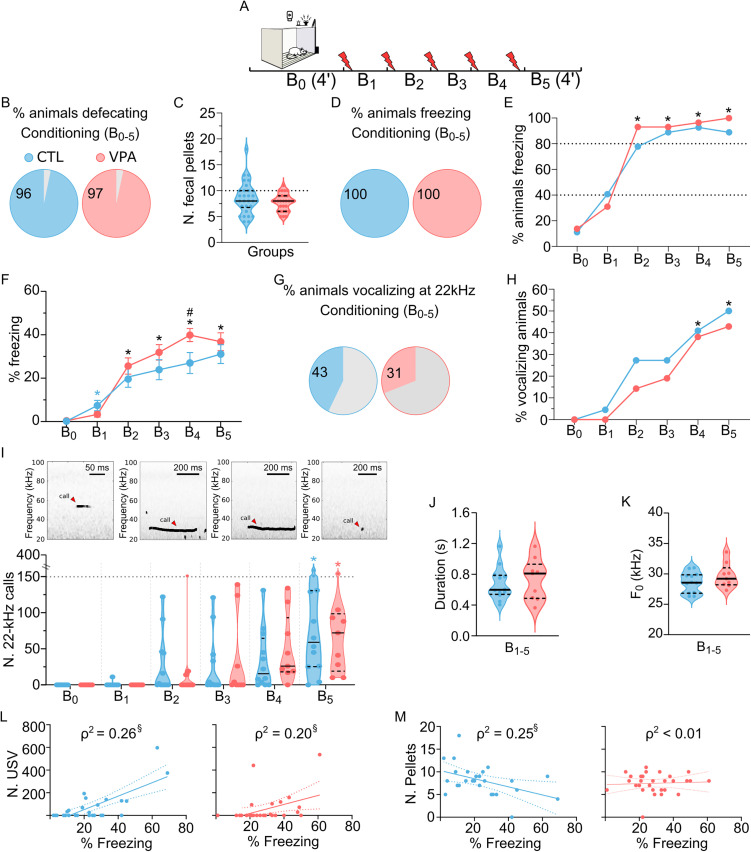
Nociceptive stress responses during fear conditioning. **(A)** Schematic illustration of the fear conditioning protocol. Following 4-min of free exploration, animals received five electrical shock stimuli. After the final stimulus, animals remained in the chamber for an additional 4 min of free exploration. **(B)** Percentage of animals releasing fecal pellets (*p* > 0.050). **(C)** Number of fecal pellets released (*p* > 0.050). **(D)** Percentage of animals displaying freezing behavior across all experimental blocks (*p* > 0.050). **(E)** Percentage of animals displaying freezing behavior in each experimental block (CTL: *p < 0.001; VPA: *p < 0.001). Dotted lines indicate the 40% and 80% reference values. **(F)** Percentage of time spent freezing in each experimental block (B4: ^#^*p* = 0.027; CTL: *p ≤ 0.008; VPA: *p < 0.001). **(G)** Percentage of animals emitting ultrasonic vocalizations (USVs) (*p* > 0.050). **(H)** Percentage of animals emitting USVs in each experimental block (CTL: *p ≤ 0.024; VPA: *p ≤ 0.047). **(I)** USVs (*calls*) recorded during the experiment. Number of calls emitted per experimental block by vocalizing animals (CTL: *p = 0.006; VPA: *p = 0.021). Dashed vertical lines delimit experimental blocks. **(J)** Median call duration during blocks B1–B5 (*p* > 0.050). **(K)** Principal frequency (F_0_) of calls emitted during blocks B1–B5 (*p* > 0.050). **(L)** Spearman correlation (*ρ*) between relative freezing behavior and the number of long 22-kHz calls emitted by CTL and VPA-treated animals (§*p* < 0.05). Each point represents one individual animal. The trend line indicates the direction and strength of the association between behaviors. **(M)** Spearman correlation between relative freezing behavior and the number of fecal pellets released by CTL and VPA-treated animals (§*p* < 0.01). Each point represents one individual animal. Panels B, D–E, G–H: Fisher’s exact test. Panel C: Mann–Whitney test. Panel F: Student’s *t*-test and repeated-measures one-way ANOVA. Panel I: Mann–Whitney and Friedman tests. Panels J–K: Mann–Whitney test. Panels L–M: Spearman tes*t*. Panel B-C: nCTL = 31 (15 females, 16 males); nVPA = 30 (14 females, 16 males). Panels D–F: nCTL = 27 (13 females, 14 males); nVPA = 29 (14 females, 15 males). Panel G-H: nCTL = 28 (13 females, 15 males); nVPA = 29 (14 females, 15 males). Panel I: nCTL = 12 (7 females, 5 males); nVPA = 9 (5 females, 4 males). Panels J–K: nCTL = 12 (7 females, 5 males); nVPA = 9 (5 females, 4 males). Panels L–M: nCTL = 26 (13 females, 13 males); nVPA = 29 (14 females, 15 males). * indicates significant within-group differences compared with block B0; Colors refer to CTL (blue), VPA (red) and both CTL and VPA groups (black). ^#^ indicates significant between-group differences.

CTL and VPA-treated rats showed comparable prevalence of defecation behavior during fear conditioning (Fig 3B), and no significant group differences were observed in the number of fecal pellets released by responsive animals (Fig 3C). Likewise, freezing behavior was similarly prevalent in both groups (Fig 3D and 3E).

Despite similar prevalence, the temporal dynamics of freezing differed between groups. CTL rats displayed increased freezing behavior from B1 onward relative to baseline (B0), whereas VPA-treated rats exhibited a delayed increase beginning only at B2 (Fig 3F). In addition, freezing responses reached greater magnitudes in VPA-treated rats, peaking at B4, where freezing levels significantly exceeded those observed in CTL rats. These findings suggest altered temporal organization and regulation of defensive responses in VPA-treated animals.

The prevalence of animals emitting ultrasonic vocalizations (USVs) did not differ significantly between groups, either globally or across experimental blocks (Fig 3G and 3H). Both groups exhibited increased prevalence of USV emission from B4 onward relative to B0. Among vocalizing animals, no significant between-group differences were observed in the total number of 22-kHz calls (Fig 3I). However, both groups showed increased call production at B5 relative to baseline.

No group differences were detected in mean call duration or fundamental frequency (F_0_; Fig 3J and 3K). Nevertheless, vocalizations emitted by VPA-treated rats displayed a bimodal distribution of call duration, characterized by clusters of shorter (~500 ms) and longer (~800 ms) calls, whereas CTL rats exhibited a more unimodal distribution centered around ~600 ms. Additionally, a subset of VPA-treated rats produced calls with F_0_ values above the third quartile of the CTL distribution (22%; > 30 kHz).

Because freezing behavior, 22-kHz ultrasonic vocalizations (USVs), and defecation are commonly used indicators of fear- and stress-related responses, we examined the relationships among these variables. Animals were considered responsive if they exhibited at least one freezing episode (≥ 3 s), regardless of whether they emitted ultrasonic vocalizations or released fecal pellets. Significant positive correlations between freezing behavior and USV emission were observed in both CTL and VPA-treated rats (Fig 3L). In contrast, freezing behavior and fecal pellet release were negatively associated in CTL animals but not in VPA-treated animals (Fig 3M).

To further evaluate this relationship, we analyzed the complete dataset (27 CTL and 29 VPA animals) using a linear mixed-effects model including group and sex as fixed effects and litter as a random intercept to account for litter-related non-independence. The analysis confirmed a significant association between freezing and USV emission (F(1,51) = 23.92, p < 0.001), with no significant Group × Freezing interaction (F(1,51) = 0.79, p > 0.05), which indicates that the correlation between the variables do not depend on the treatment. Consistent with this interpretation, correlation showed that although significant positive correlations were detected in CTL (ρ = 0.51, p = 0.007) and VPA groups (ρ = 0.45, p = 0.014), there was no difference between them. In contrast, freezing behavior and fecal pellet release showed a significant negative association (F(1,51) = 10.63, p = 0.002) that was primarily driven by CTL animals (CTL: ρ = −0.502, p = 0.008; VPA: ρ = −0.038, p = 0.846). Moreover, a significant Group × Freezing interaction was observed (F(1,51) = 5.05, p = 0.029), indicating that the relationship between freezing behavior and defecation differed between CTL and VPA-treated animals. Specifically, freezing behavior was negatively associated with pellet release in CTL animals, whereas this relationship was markedly attenuated in VPA-treated animals.These observations further support our previous results.

Memory retrieval assessed 24 h after conditioning revealed no significant differences between groups in either freezing prevalence or freezing magnitude (S4 Fig). Both groups exhibited freezing levels above baseline, indicating preserved retrieval of the conditioned response. No sex-related differences were detected.

### Prenatal exposure to VPA promotes delayed fear response during emotional contagion

**Fig 4:**
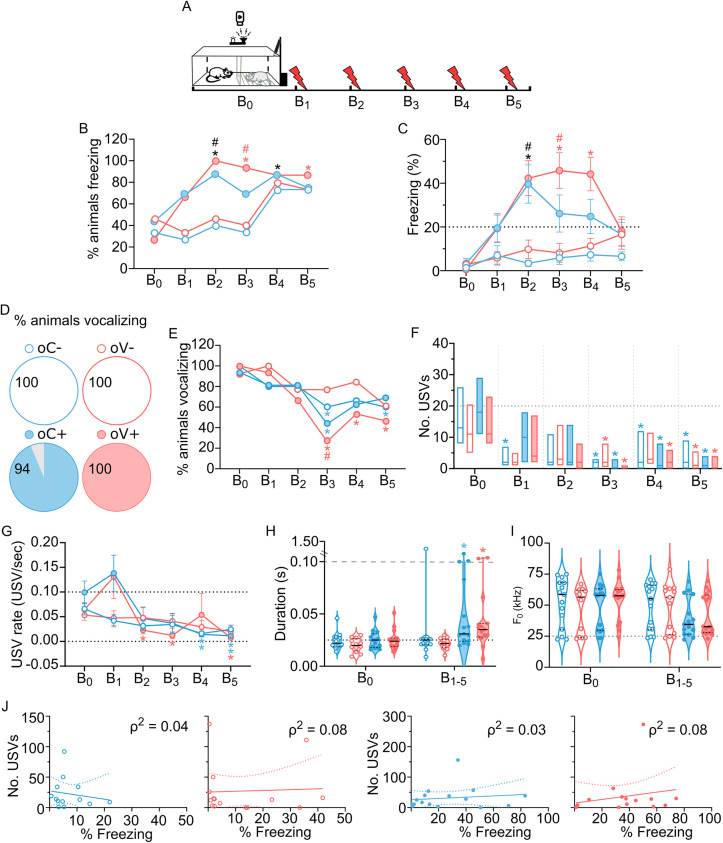
Behavioral analysis during social stress. **(A)** Schematic illustration of the experimental design. Following a 4-min period of free exploration, the demonstrator received five electrical shock stimuli. After the final stimulus, the animals remained in the chamber for an additional 4 min of free exploration. **(B)** Percentage of observer animals displaying freezing behavior in each experimental block (^#^*p* ≤ 0.030; oC + : *p = 0.038; oV + : *p ≤ 0.007). **(C)** Percentage of time spent freezing in each experimental block (^#^*p* ≤ 0.027; oC + : *p = 0.006; oV + : *p = 0.002). The dotted line indicates the 20% reference value. **(D)** Percentage of observer animals vocalizing (all frequencies combined) across the entire experiment (*p* > 0.050). **(E)** Percentage of animals vocalizing in each experimental block (^#^*p* = 0.046; oC-: *p = 0.041; oC + : *p = 0.025; oV + : *p ≤ 0.025). **(F)** Number of calls emitted in each experimental block (^#^*p* > 0.050; oC-: *p ≤ 0.024; oV-: *p ≤ 0.011; oC + : *p = 0.027; oV + : *p ≤ 0.011). Dotted horizontal lines indicate the reference values of 20 calls. Dashed vertical lines delimit experimental blocks. **(G)** Call rate in each experimental block (^#^*p* > 0.050; oC-: *p ≤ 0.024; oC + : *p = 0.008; oV + : *p ≤ 0.009). **(H)** Median call duration during B0 and B1–B5. The dashed line indicates the 0.1-s reference value. **(I)** Principal frequency of calls during B0 and B1–B5. The dashed line indicates the 25kHz reference value. **(J)** Correlation between freezing behavior and number of vocalizations (all frequencies combined) emitted by observer animals. Each point represents one individual animal. Panel B and E: Chi-square. Panels C and G: repeated-measures two-way ANOVA. Panel D: Fisher’s exact test. Panel F: Kruskal–Wallis and Friedman tests. Panels H and I: Kruskal–Wallis and Wilcoxon tests. Panel J: Correlation analysis (linear mixed model and Spearman correlation, *ρ > 0.050*) between behavior outputs. Panel B: noC− = 15 (7 females, 8 males); noV− = 15 (7 females, 8 males); noC+ = 16 (8 females, 8 males); noV+ = 15 (8 females, 7 males). Panel C and J: noC− = 13 (5 females, 8 males); noV− = 15 (7 females, 8 males); noC+ = 16 (8 females, 8 males); noV+ = 15 (8 females, 7 males). Panels D-I: noC− = 15 (7 females, 8 males); noV− = 13 (6 females, 7 males); noC+ = 16 (8 females, 8 males); noV+ = 15 (8 females, 7 males). ^#^ indicates significant differences between Shock (+) and non-Shock (−) groups; * indicates significant within-group differences compared with block B0. Colors refer to CTL (blue), VPA (red) and both CTL and both CTL and VPA groups (black).

During the emotional contagion paradigm, observer (OBS) animals were exposed to demonstrator (DEM) rats that either received foot shocks or remained unshocked (Fig 4A). Observation of distressed DEM animals increased freezing behavior in both CTL and VPA-treated observers (oC+ and oV+), whereas no comparable effect was observed in animals exposed to non-shocked demonstrators (oC− and oV−).

Between-group comparisons showed that, at B2 there was a significantly larger engagement in freezing of oC+ animals compared to oC-, as well as of oV+ animals compared to oV-. Notably, in the oV+ group, this elevated freezing response persisted through B3 (Fig 4B). In addition, within-group analysis (compared to B0), a significantly higher prevalence of freezing behavior was observed in the oC+ group at B2 and B4 and in the oV+ group at B2-B5. We also observed an increase in the relative number of animals expressing freezing behavior at B4-B5 in oC- and oV- groups, but they were not statistically significant.

When comparing the freezing behavior of responsive animals (i.e., those exhibiting any freezing) at B2, both oC+ and oV+ rats displayed significantly higher levels of freezing compared to their respective CTLs (oC- and oV-). Notably, the difference between oV+ and oV- rats persisted through B3 (Fig 4C). Within-group analysis revealed that both oC+ and oV+ animals exhibited increased freezing in response to witnessing repeated shocks administered to their DEM partner, relative to B0. This increase reached statistical significance starting at B2. Notably, while the heightened freezing in oC+ rats was restricted to B2, oV+ rats maintained an elevated freezing response from B2 through B4.

Linear mixed-effects analyses including litter as a random effect were performed to account for potential non-independence among animals from the same litter. This analysis revealed a highly significant Treatment × Block interaction (F(5,352) = 10.729, p = 1.30 × 10 − 9), indicating that freezing dynamics across experimental blocks differed as a function of treatment condition. Importantly, the estimated litter intraclass correlation coefficient was extremely small: ICC_Litter = 0.0069 indicating that less than 1% of the total variance was attributable to litter of origin, i.e., animals from the same litter behaved almost independently with respect to the analyzed outcome. In addition, as a conservative sensitivity analysis, we averaged freezing values within litters and reanalyzed the data using litter-level observations. Despite the substantial reduction in statistical power resulting from litter averaging, the Treatment × Block interaction remained statistically significant (F(5,88) = 3.078, p = 0.013). Moreover, the estimated litter ICC in this conservative analysis was effectively zero, demonstrating that the reported effects were robust to litter-based clustering and not attributable to pseudo-replication.

The prevalence of vocalizing animals was similar across groups (Fig 4D and 4E), although shock exposure progressively reduced vocal engagement in observer animals. Relative to baseline, oC + , oV + , and oC− rats showed reduced vocalization prevalence, with effects emerging earlier and persisting longer in oV+ animals.

Changes in the number of vocalizations produced by OBS animals were also observed compared to the B0, with no differences between groups (Fig 4F). Among responsive animals, a reduction in vocal behavior was observed from B3 onward in the oC+ and oV + , as well as in oV- groups. In contrast, the oC- animals exhibited a significant decrease as early as B1, relative to B0. Analysis of vocalization rate (USVs per minute) revealed that oV+ animals exhibited a consistent reduction during sessions B2, B3, and B5. In contrast, both oC+ and oC- groups showed a decline in vocal behavior only from session B4 onward. The oV- group maintained a stable vocalization rate across all sessions. Notably, both oC+ and oV+ animals displayed a peak in vocalization rate during session B1, followed by a progressive decline in subsequent blocks. (Fig 4G).

Analysis of acoustic properties revealed longer call durations in oC+ and oV+ animals during B1-B5 relative to baseline (Fig 4H). In contrast, no differences in fundamental frequency were detected either across blocks or between groups (Fig 4I).

Correlation analysis between freezing and vocal production during the emotional contagion in responsive animals showed no significant differences (Fig 4J). Similar results were observed when we analyzed all experimental animals (31 CTL animals: 15 oC− and 16 oC + ; 30 VPA animals: 15 oV− and 15 oV+). Linear mixed-effects analyses revealed no significant association between freezing behavior and USV emission (F(1,55) = 2.37, p = 0.129), and no significant Group (F(1,55) = 2.2 × 10^−5^, p = 0.99) or Treatment effects (F(1,55) = 0.033, p = 0.857). Consistent with these findings, Spearman correlation analyses within each subgroup revealed no statistically significant associations between freezing behavior and USV production (oC − : ρ = −0.1898; oC + : ρ = 0.1740; oV − : ρ = −0.2796; oV + : ρ = 0.2806; all p > 0.05). Together, these results indicate no detectable relationship between freezing behavior and USV production during emotional contagion in either CTL or VPA-treated animals. No sex-related differences were observed in the susceptibility and magnitude of the freezing and vocal responses (S5 Fig).

Because observer responses depend on the behavior of demonstrator animals, we additionally quantified freezing and vocalizations emitted by DEM rats (S6 Fig). Shock exposure robustly increased freezing prevalence and freezing magnitude in both dC+ and dV+ groups relative to non-shocked controls, with no differences between CTL and VPA-treated demonstrators. Likewise, both dC+ and dV+ animals exhibited marked increases in USV prevalence and call production following shock exposure. Acoustic properties of demonstrator calls remained similar between groups. Together, these findings indicate that CTL and VPA-treated observers were exposed to comparable social distress cues.

## Discussion

Animal models remain essential for elucidating the mechanisms underlying emotional dysregulation in neurodevelopmental disorders and for developing therapeutic strategies. In the present study, we systematically investigated stress responsiveness across multiple behavioral paradigms and emotional contagion responses in a rat model of autism induced by prenatal exposure to VPA.

Across the behavioral paradigms employed, VPA-treated rats exhibited a consistent pattern of altered stress responsiveness characterized by delayed habituation, prolonged defensive reactions, and atypical coordination among behavioral and autonomic responses. During repeated handling and electro-tactile stimulation, VPA-treated animals showed increased susceptibility to stress-related responses, reflected by enhanced defecation behavior despite preserved tactile and nociceptive thresholds. During fear conditioning, VPA-treated rats displayed delayed onset and greater magnitude of freezing responses while maintaining overall levels of ultrasonic vocalization comparable to CTL animals. Correlation analyses further indicated altered relationships among freezing, defecation, and vocal behavior in VPA-treated animals, suggesting disrupted coordination of emotional and physiological stress responses. In the emotional contagion paradigm, VPA-treated observer rats exhibited prolonged freezing and altered vocal dynamics in response to witnessing a distressed conspecific, whereas demonstrator animals showed largely comparable behavioral outputs across groups. Together, these findings indicate that prenatal exposure to VPA does not simply enhance stress reactivity, but rather alters the temporal organization, habituation, and integration of fear- and stress-related responses across both nonsocial and social contexts.

Unfamiliar handling in rodents is known to elicit stress-related behaviors such as avoidance and increased defecation, which typically attenuate with repeated exposure to the experimenter [39–41]. In the present study, the greater number of VPA-treated animals releasing fecal pellets, as well as the greater number of fecal pellets released during the first experimental days, compared to CTL, suggest an increased susceptibility to stress responses to innocuous stimuli. Furthermore, although both VPA-treated and CTL animals showed a reduction in fecal pellet production after four days of handling, suggesting successful habituation responses in both groups, the delayed reduction observed in VPA-treated rats indicates a slower adaptation to nonthreatening stimuli, consistent with reports of atypical sensory processing, stress management, and impaired desensitization in individuals with ASD. Evidence from human studies consistently demonstrates altered responses to tactile stimuli in ASD, characterized by both hyper- and hypo-behavioral reactivity, but with hyper-reactivity being the most consistently observed phenotype [42–49]. These behavioral manifestations suggest autonomic nervous system (ANS) dysregulation. This alteration is accompanied by elevated baseline electrodermal activity and heart rate, corroborating the dysregulation of the ANS [13–15]. A recent study examining ANS responses to affective touch (e.g., gentle stroking of hairy skin) in children reported smaller autonomic reactivity in those with ASD [13]. Similarly, the increased defecation response of VPA-treated rats during early handling sessions may reflect analogous autonomic dysregulation and an elevated susceptibility for stress responses to innocuous stimuli.

Although a greater proportion of VPA-treated animals exhibited defecation in response to electro-tactile stimulation compared to CTLs, suggesting increased susceptibility, neither the freezing behavior nor the number of fecal pellets differed significantly between groups, indicating comparable magnitudes of stress responses. These findings are consistent with our observations of tactile and nociceptive thresholds and align with previous studies demonstrating preserved nociceptive thresholds to electrical stimulation in VPA-exposed rats [50]. Notably, prior investigations into tactile sensitivity in VPA-treated animals have consistently reported increased responsiveness to mechanical allodynia and diminished sensitivity to thermal nociceptive stimuli [35,36,51–55].

During the fear conditioning paradigm, VPA and CTL animals were similarly susceptible to stress-induced defecation, freezing, and 22-kHz vocalization behaviors, following a similar time-course in both groups. Although studies examining the dynamics of the freezing response in VPA-treated animals remain limited, our findings are consistent with a previous report showing increased shock-induced freezing in VPA-treated rats relative to CTLs [56]. In contrast, other studies assessing overall freezing across the entire experimental session have reported preserved freezing behavior in VPA-treated animals [36,50,57]. These discrepancies may arise from variations in experimental design, strain-specific factors, or developmental stage differences. Further analysis of our data indicated a delayed onset of freezing behavior in the VPA group, suggesting impairments in the integration or coordination of stress-related emotional responses, including sensory processing, decision-making, motor planning and execution, or disruptions in emotional and motivational regulation. This delayed response, alongside preserved vocalization, is consistent with the hypothesis of altered integration or coordination of emotional processing under stress. Moreover, the observed negative correlation between freezing behavior and fecal pellet release—along with the progressive increase in freezing observed in CTL animals—suggests that defecation predominates at lower stress intensities, whereas freezing becomes more prominent as stress levels escalate. The lack of this correlation in VPA-treated rats provides additional evidence for a disruption in the integration of physiological responses associated with emotional stress.

During fear retrieval, behavioral responses were limited, particularly for vocalizations and defecation, restricting the analysis to freezing behavior. No group differences were observed in freezing magnitude or incidence. These findings are consistent with prior reports indicating preserved retrieval responses in VPA-treated rats and further suggest that acute associative memory of the conditioned stimulus is intact in this model.

Nociceptive responses in individuals with autism spectrum disorder (ASD) vary across developmental stages. Human studies have shown that children with ASD often exhibit hypersensitivity to pressure and cold pain, while adolescents tend to display thermal pain thresholds within the normative range. In adults, thermal pain thresholds are preserved, but pain ratings are elevated and more variable [58–65]. These developmental discrepancies in pain sensitivity further underscore the complexity and heterogeneity of sensory and stress-related phenotypes in ASD. Our findings—demonstrating preserved nociceptive sensitivity but altered stress habituation in adolescent rats—parallel this developmental trajectory and suggest translational relevance for the VPA model in this domain.

In the emotional contagion paradigm, both CTL and VPA observer rats exhibited freezing and 22-kHz vocalizations in response to a distressed conspecific, consistent with prior findings in CTL animals [66,67]. However, VPA observers displayed prolonged freezing and delayed habituation, suggestive of increased sensitivity to social distress or impairments in social buffering mechanisms. Notably, demonstrator animals showed comparable freezing behavior across groups (S6B and S6C Fig) and only minor differences in vocalizations (S6D–S6F Fig), indicating that the sensory cues available to observers were largely equivalent. Additionally, the acoustic properties of demonstrator calls were similar across conditions, supporting the conclusion that CTL and VPA observers were exposed to comparable emotional signals (S6G and S6H Fig). Although we cannot entirely exclude the possibility that subtle differences in auditory sensitivity contribute to the observed behavioral variations between VPA-treated and control animals, our previous research indicates a difference of less than 5% in cortical sensitivity for high-frequency sounds. Specifically, high-density cortical recordings showed that 36% of A1 sites in VPA-treated rats were responsive to high-frequency (10–50 kHz), low-intensity (<50 dB) stimuli, compared to 40% in control animals [68]. Together, these results indicate that VPA-treated animals exhibit altered habituation and heightened responsiveness to social distress, reflecting a dysregulated emotional contagion response. This phenotype underscores the utility of the VPA model for investigating stress reactivity and social emotion regulation deficits relevant to ASD.

While emotional contagion has been well-characterized in various rodent strains, to our knowledge, no previous studies have examined this phenomenon in a rodent model of autism. Our findings thus provide novel evidence that, despite their established social deficits, juvenile VPA-treated animals still perceive social stressors as salient, although the integration or coordination of their behavioral responses to these stimuli appears to be altered. In addition, the preserved sensitivity to social cues but fail to downregulate stress responses following social exposure of VPA-treated animals results are consistent with human data, where both children with ASD and TD peers show increased cortisol levels in response to social interaction. However, while TD children demonstrate cortisol habituation by the end of the exposure, this attenuation is absent in children with ASD. Additionally, among children exhibiting the highest cortisol reactivity—including both ASD and TD groups—those with ASD engaged in lower levels of social interaction. Complementary findings from the same research group further report that elevated cortisol responses to social challenges are positively associated with increased daily stress and sensory processing difficulties [69–72]. This pattern underscores the translational value of the VPA model for investigating stress regulation abnormalities in ASD. By capturing both behavioral and physiological features of impaired social stress adaptation, the model offers an important platform for elucidating the underlying neurobiological mechanisms and for identifying potential targets for therapeutic intervention.

Notably, across all experimental paradigms, VPA-treated animals consistently exhibited a trend toward increased involuntary responses (defecation and freezing) and slightly reduced voluntary vocalizations relative to CTL animals, even in the absence of statistically significant group differences.

Sex differences in stress responsiveness were not observed across any of the paradigms employed. This aligns with previous research demonstrating that juvenile rats exposed to VPA do not exhibit significant sex-based differences in social play behavior [73]. These findings are consistent with a human study in which VPA-exposed ASD children have a much reduced autism male bias (1.2–2.4:1) compared to non-VPA-exposed ASD children (3.3:1) [74,75]. One possible explanation for the attenuated sex differences is that VPA acts as a neurodevelopmental risk factor whose effects may override or bypass sex-dependent protective mechanisms. Alternatively, it has been proposed that females with ASD may engage in compensatory social behaviors that mask symptomatology, contributing to diagnostic underrepresentation and an artificially reduced prevalence [76–79]. The absence of sex effects in our study may also reflect limitations in the sensitivity of the employed behavioral assays to detect subtle sex-dependent phenotypic variations or may result from the homogeneity of the VPA-induced phenotype across sexes. To better elucidate sex-specific mechanisms, future studies should incorporate hormonal profiling or circuit-level analyses and more nuanced behavioral assessments designed to detect subtle, sex-specific traits.

Some limitations of the present study include the absence of auditory assessments, which would be important to control for individual variability in hearing-dependent fear responses, the lack of corticosterone measurements, which could have provided additional insight into possible HPA-axis alterations associated with the behavioral phenotypes observed in VPA-treated animals and the indirect validation of emitter identity during the emotional contagion sessions. These limitations should be addressed in future studies to further clarify the mechanisms underlying the behavioral phenotypes described here.

## Conclusion

VPA-treated rats exhibited altered stress and fear-related behaviors across multiple paradigms. Compared to CTLs, they showed heightened and prolonged physiological responses to handling and electro-tactile stimulation, as well as delayed and exaggerated freezing during fear conditioning. Our findings also provide evidence that animals prenatally exposed to VPA have altered coordination between freezing response and autonomic visceral control. During emotional contagion, VPA-treated observers demonstrated more sustained freezing and earlier reductions in vocalization when witnessing a conspecific in distress, suggesting altered emotional processing. These findings support a profile of increased emotional reactivity and impaired integration of fear-related responses in VPA-treated animals.

## Supporting information

S1 Table Summary of animals and litters used across experiments. Offspring from multiple litters were distributed across experimental conditions to minimize potential confounding effects of litter identity. Within each experimental paradigm, independent litters were assigned to the CTL and VPA groups, such that no litter contributed animals to both treatment conditions.
(TIFF)

S1 Fig Assignment of observer (OBS) and demonstrator (DEM) vocalizations. Vocalizations produced during the emotional contagion paradigm were assigned to DEM or OBS animals based on ultrasonic vocalization (USV) power intensity. Thresholds were established experimentally (A–C) by comparing the power of USVs recorded with a microphone positioned either in the same chamber as the vocalizing animal or in the opposite chamber. (A–B) Mean USV power during maternal separation at postnatal days P08 and P14 (^#^*p* < 0.0001). (C) Mean USV power during fear conditioning at P35–P38 (^#^*p* < 0.0001). Mann–Whitney test. Panel A: *n*USV_opposite = 41; *n*USV_microphone = 35 (*N* = 1). Panel B: *n*USV_opposite = 55; *n*USV_microphone = 69 (*N* = 1). Panel C: *n*USV_opposite = 153; *n*USV_microphone = 153 (*N* = 6). Vocalizations with power > −76 dB were classified as originating from animals located in the microphone chamber (DEM chamber), whereas vocalizations <−85 dB were classified as originating from animals in the opposite chamber (OBS chamber). Vocalizations with power values between −76 and −85 dB were excluded from analyses to minimize ambiguity in chamber assignment. Calls within this interval represented 9.8% of all vocalizations emitted during the emotional contagion experiment (2086/21388 calls), corresponding to 8.4% of CTL calls and 11.2% of VPA calls.
(TIFF)

S2 Fig Sex-related differences in touch-induced defecation responses. (A) Schematic illustration of the touch sensitivity experimental design. (B) Number of fecal pellets released on each experimental day by female and male CTL animals (p > 0.050). (C) Number of fecal pellets released on each experimental day by female and male VPA-treated animals (p > 0.050). Mann–Whitney test, CTL: *n* = 18 (9 females, 9 males); VPA: *n* = 21 (10 females, 11 males).
(TIFF)

S3 Fig Tactile and nociceptive thresholds. (A) Tactile and nociceptive thresholds (^#^*p* > 0.050; *p < 0.001). (B) Tactile thresholds in female and male animals (^&^*p* = 0.007). Panel A: paired Student’s *t*-test. Panel B: Mann–Whitney test. Panel A and B: nCTL = 30 (15 females, 15 males); nVPA = 28 (15 females, 13 males). ^&^ indicates significant sex-related differences.
(TIFF)

S4 Fig Fear memory retrieval. (A) Schematic illustration of the experimental design. Rats were placed in the center of the apparatus facing one of the metal walls and remained in the chamber for 5 min before being returned to their home cage. (B) Percentage of animals displaying freezing behavior during fear conditioning memory retrieval (p > 0.05). (C) Percentage of time spent freezing during the fear conditioning baseline (B0) and memory retrieval session (*p* < 0.001). Panel B: Fisher’s exact test. Panel C: Student’s *t*-test and paired Student’s *t*-test, CTL: *n* = 27 (13 females, 14 males); VPA: *n* = 28 (14 females, 14 males). * indicates significant within-group differences.
(TIFF)

S5 Fig Sex-related differences in emotional contagion freezing responses. (A) Schematic illustration of the emotional contagion experimental design. (B) Percentage of time spent freezing in each experimental block by female and male CTL animals. (C) Percentage of time spent freezing in each experimental block by female and male VPA-treated animals. The dotted line indicates the 20% reference value. Repeated-measures two-way ANOVA, NoC + : *n* = 16 (8 females, 8 males); NoV + : *n* = 15 (8 females, 7 males).
(TIFF)

S6 Fig Behavioral and vocal responses of DEM animals during emotional contagion. (A) Schematic illustration of the emotional contagion experimental design. Following a 4-min period of free exploration, DEM animals received five electrical shock stimuli. After the final stimulus, animals remained in the chamber for an additional 4 min of free exploration. (B) Percentage of animals displaying freezing behavior in each experimental block (CTL: ^#^*p* ≤ 0.011; VPA: ^#^*p* ≤ 0.021; dC + : *p < 0.001; dV + : *p ≤ 0.012). (C) Percentage of time spent freezing by animals in each experimental block (CTL: ^#^*p* ≤ 0.008; VPA: ^#^*p* < 0.001; dC + : *p < 0.001; dV + : *p ≤ 0.022). (D) Percentage of animals emitting USVs across the experiment (B0–B5) (CTL: ^#^*p* < 0.001; VPA: ^#^*p* ≤ 0.011). (E) Percentage of animals emitting USVs in each experimental block (CTL: ^#^*p* ≤ 0.002; VPA: ^#^*p* ≤ 0.006; dC + : *p ≤ 0.042; dV + : *p ≤ 0.027). (F) Number of USVs emitted in each experimental block (CTL: ^#^*p* ≤ 0.017; VPA: ^#^*p* ≤ 0.024; dV − : *p = 0.017; dC + : *p ≤ 0.001; dV + : *p ≤ 0.042). (G) Duration of USVs emitted during blocks B1–B5 (p > 0.050). (H) Principal frequency of USVs emitted during blocks B1–B5 (p > 0.050). Panels B, D, and E: Fisher’s exact test. Panel C: repeated-measures two-way ANOVA. Panel F: Friedman test. Panels G–H: Mann–Whitney test. Panels B–E: ndC− = 15 (7 females, 8 males); ndV− = 15 (7 females, 8 males); ndC+ = 16 (8 females, 8 males); ndV+ = 15 (8 females, 7 males). Panel F: noC− = 5 (2 females, 3 males); noV− = 6 (2 females, 4 males); noC+ = 16 (8 females, 8 males); noV+ = 14 (8 females, 6 males). Panels G–H: noC+ = 16 (8 females, 8 males); noV+ = 14 (8 females, 6 males). ^#^ indicates significant differences between Shock (+) and non-Shock (−) groups; * indicates significant within-group differences compared with block B0. Because the behavioral responses of observer (OBS) rats are expected to depend on the behavior of demonstrator (DEM) rats, we quantified freezing behavior and ultrasonic vocalizations (USVs) emitted by DEM animals during the emotional contagion paradigm. As expected, foot shock exposure significantly increased the prevalence of freezing behavior in both shocked groups (dC+ and dV+) compared to their respective non-shocked controls (dC- and dV-), regardless of prenatal treatment (S6A and S6B Fig). No significant differences in freezing prevalence were found between dV+ and dC+ animals or between dV- and dC- animals.Among responsive individuals, both the dC+ and dV+ groups exhibited a sharp, significant increase in freezing time following the onset of shock stimuli (S6C Fig). Specifically, compared to baseline (B0) levels, dC+ and dV+ animals increased their freezing behavior at block B1, reaching a plateau sustained from B2 to B5. Conversely, dV- and dC- controls showed no deviations from B0 freezing levels. Crucially, no significant differences in freezing duration were detected in between-group comparisons (dC + vs. dV+ or dC- vs. dV-; p > 0.05).Shocked demonstrators also showed a higher prevalence of vocalization compared to non-shocked controls (S6D Fig). Analysis of vocal engagement across experimental blocks revealed: (1) no significant differences between dV + vs. dC+ or between dV- vs. dC-; (2) significant differences between dV + vs. dV (beginning at block B2) and between dC + vs. dC- (beginning at block B1); and (3) a significant increase in vocalization prevalence relative to B0 in both dV+ and dC+ groups from block B2 onward (S6E Fig). Control dV- and dC- rats maintained baseline-like vocal engagement throughout the experiment.In terms of call volume, DEM rats exhibited robust USV emission following foot shock exposure. While the total number of emitted USVs did not differ significantly between the dV+ and dC+ groups, both shocked groups demonstrated a marked increase in call numbers from block B4 onward relative to their respective non-shocked controls (dC- and dV-; S6F Fig). Furthermore, relative to baseline (B0), a significant increase in vocal production was observed in dV+ (blocks B2–B5), dC+ (blocks B2 and B4–B5), and dV- (block B1) animals. No significant within-group changes over time were found for the dC- controls. Finally, bioacoustic properties—namely call duration and principal frequency (F_0_)—did not differ between dV+ and dC+ animals (S6G and S6H Fig). Due to the low number of vocalizations emitted by dV- and dC- animals (median of 3 calls per block), their acoustic properties were excluded from further statistical analysis.
(TIFF)
