# Correction: Altered stress and fear responses in the VPA rat model of autism: Behavioral dissociation across tactile, nociceptive, and social contexts

**DOI:** 10.1371/journal.pone.0357445

**Published:** 2026-09-01

**Authors:** Debora Hashiguchi, Ana Luiza Dias, Rodrigo Neves Romcy-Pereira

There are errors in the Funding statement. The correct Funding statement is as follows: This work was supported by the Coordenação de Aperfeiçoamento de Pessoal de Nível Superior (CAPES, Brazil; #88882.376056/2019–1 to DH), partially by the Conselho Nacional de Desenvolvimento Científico e Tecnológico (CNPq, #383539/2025–0 to DH), and by CNPq (#010186/2018–2 and #421955/2023–6 to RNRP).

S1 Table and S1 to S6 Figs were uploaded incorrectly. Please see the correct S1 Table and S1 to S6 Figs here.

## Supporting information

S1 Table Summary of animals and litters used across experiments. Offspring from multiple litters were distributed across experimental conditions to minimize potential confounding effects of litter identity. Within each experimental paradigm, independent litters were assigned to the CTL and VPA groups, such that no litter contributed animals to both treatment conditions.
(TIFF)

S1 Fig Assignment of observer (OBS) and demonstrator (DEM) vocalizations. Vocalizations produced during the emotional contagion paradigm were assigned to DEM or OBS animals based on ultrasonic vocalization (USV) power intensity. Thresholds were established experimentally (A–C) by comparing the power of USVs recorded with a microphone positioned either in the same chamber as the vocalizing animal or in the opposite chamber. (A–B) Mean USV power during maternal separation at postnatal days P08 and P14 (^#^*p*  <  0.0001). (C) Mean USV power during fear conditioning at P35–P38 (^#^*p*  <  0.0001). Mann–Whitney test. Panel A: *n*USV_opposite = 41; *n*USV_microphone  =  35 (*N*  =  1). Panel B: *n*USV_opposite = 55; *n*USV_microphone  =  69 (*N*  =  1). Panel C: *n*USV_opposite = 153; *n*USV_microphone  =  153 (*N*  =  6). Vocalizations with power  > −76 dB were classified as originating from animals located in the microphone chamber (DEM chamber), whereas vocalizations <−85 dB were classified as originating from animals in the opposite chamber (OBS chamber). Vocalizations with power values between −76 and −85 dB were excluded from analyses to minimize ambiguity in chamber assignment. Calls within this interval represented 9.8% of all vocalizations emitted during the emotional contagion experiment (2086/21388 calls), corresponding to 8.4% of CTL calls and 11.2% of VPA calls.
(TIFF)

S2 Fig Sex-related differences in touch-induced defecation responses. (A) Schematic illustration of the touch sensitivity experimental design. (B) Number of fecal pellets released on each experimental day by female and male CTL animals (p  >  0.050). (C) Number of fecal pellets released on each experimental day by female and male VPA-treated animals (p  >  0.050). Mann–Whitney test, CTL: *n*  =  18 (9 females, 9 males); VPA: *n*  =  21 (10 females, 11 males).
(TIFF)

S3 Fig Tactile and nociceptive thresholds. (A) Tactile and nociceptive thresholds (^#^*p*  >  0.050; *p  <  0.001). (B) Tactile thresholds in female and male animals (^&^*p*  =  0.007). Panel A: paired Student’s *t*-test. Panel B: Mann–Whitney test. Panel A and B: nCTL  =  30 (15 females, 15 males); nVPA  =  28 (15 females, 13 males). ^&^ indicates significant sex-related differences.
(TIFF)

S4 Fig Fear memory retrieval. (A) Schematic illustration of the experimental design. Rats were placed in the center of the apparatus facing one of the metal walls and remained in the chamber for 5 min before being returned to their home cage. (B) Percentage of animals displaying freezing behavior during fear conditioning memory retrieval (p  >  0.05). (C) Percentage of time spent freezing during the fear conditioning baseline (B0) and memory retrieval session (*p*  <  0.001). Panel B: Fisher’s exact test. Panel C: Student’s *t*-test and paired Student’s *t*-test, CTL: *n*  =  27 (13 females, 14 males); VPA: *n*  =  28 (14 females, 14 males). * indicates significant within-group differences.
(TIFF)

S5 Fig Sex-related differences in emotional contagion freezing responses. (A) Schematic illustration of the emotional contagion experimental design. (B) Percentage of time spent freezing in each experimental block by female and male CTL animals. (C) Percentage of time spent freezing in each experimental block by female and male VPA-treated animals. The dotted line indicates the 20% reference value. Repeated-measures two-way ANOVA, NoC  +  : *n*  =  16 (8 females, 8 males); NoV  +  : *n*  =  15 (8 females, 7 males).
(TIFF)

S6 Fig Behavioral and vocal responses of DEM animals during emotional contagion. (A) Schematic illustration of the emotional contagion experimental design. Following a 4-min period of free exploration, DEM animals received five electrical shock stimuli. After the final stimulus, animals remained in the chamber for an additional 4 min of free exploration. (B) Percentage of animals displaying freezing behavior in each experimental block (CTL: ^#^*p*  ≤  0.011; VPA: ^#^*p*  ≤  0.021; dC  +  : *p  <  0.001; dV  +  : *p  ≤  0.012). (C) Percentage of time spent freezing by animals in each experimental block (CTL: ^#^*p*  ≤  0.008; VPA: ^#^*p*  <  0.001; dC  +  : *p  <  0.001; dV  +  : *p  ≤  0.022). (D) Percentage of animals emitting USVs across the experiment (B0–B5) (CTL: ^#^*p*  <  0.001; VPA: ^#^*p*  ≤  0.011). (E) Percentage of animals emitting USVs in each experimental block (CTL: ^#^*p*  ≤  0.002; VPA: ^#^*p*  ≤  0.006; dC  +  : *p  ≤  0.042; dV  +  : *p  ≤  0.027). (F) Number of USVs emitted in each experimental block (CTL: ^#^*p*  ≤  0.017; VPA: ^#^*p*  ≤  0.024; dV  −  : *p  =  0.017; dC  +  : *p  ≤  0.001; dV  +  : *p  ≤  0.042). (G) Duration of USVs emitted during blocks B1–B5 (p  >  0.050). (H) Principal frequency of USVs emitted during blocks B1–B5 (p  >  0.050). Panels B, D, and E: Fisher’s exact test. Panel C: repeated-measures two-way ANOVA. Panel F: Friedman test. Panels G–H: Mann–Whitney test. Panels B–E: ndC −   =  15 (7 females, 8 males); ndV −   =  15 (7 females, 8 males); ndC +   =  16 (8 females, 8 males); ndV +   =  15 (8 females, 7 males). Panel F: noC −   =  5 (2 females, 3 males); noV −   =  6 (2 females, 4 males); noC +   =  16 (8 females, 8 males); noV +   =  14 (8 females, 6 males). Panels G–H: noC +   =  16 (8 females, 8 males); noV +   =  14 (8 females, 6 males). ^#^ indicates significant differences between Shock (+) and non-Shock (−) groups; * indicates significant within-group differences compared with block B0. Because the behavioral responses of observer (OBS) rats are expected to depend on the behavior of demonstrator (DEM) rats, we quantified freezing behavior and ultrasonic vocalizations (USVs) emitted by DEM animals during the emotional contagion paradigm. As expected, foot shock exposure significantly increased the prevalence of freezing behavior in both shocked groups (dC+ and dV+) compared to their respective non-shocked controls (dC- and dV-), regardless of prenatal treatment (S6A and S6B Fig). No significant differences in freezing prevalence were found between dV+ and dC+ animals or between dV- and dC- animals.Among responsive individuals, both the dC+ and dV+ groups exhibited a sharp, significant increase in freezing time following the onset of shock stimuli (S6C Fig). Specifically, compared to baseline (B0) levels, dC+ and dV+ animals increased their freezing behavior at block B1, reaching a plateau sustained from B2 to B5. Conversely, dV- and dC- controls showed no deviations from B0 freezing levels. Crucially, no significant differences in freezing duration were detected in between-group comparisons (dC  +  vs. dV+ or dC- vs. dV-; p  >  0.05).Shocked demonstrators also showed a higher prevalence of vocalization compared to non-shocked controls (S6D Fig). Analysis of vocal engagement across experimental blocks revealed: (1) no significant differences between dV  +  vs. dC+ or between dV- vs. dC-; (2) significant differences between dV  +  vs. dV (beginning at block B2) and between dC  +  vs. dC- (beginning at block B1); and (3) a significant increase in vocalization prevalence relative to B0 in both dV+ and dC+ groups from block B2 onward (S6E Fig). Control dV- and dC- rats maintained baseline-like vocal engagement throughout the experiment.In terms of call volume, DEM rats exhibited robust USV emission following foot shock exposure. While the total number of emitted USVs did not differ significantly between the dV+ and dC+ groups, both shocked groups demonstrated a marked increase in call numbers from block B4 onward relative to their respective non-shocked controls (dC- and dV-; S6F Fig). Furthermore, relative to baseline (B0), a significant increase in vocal production was observed in dV+ (blocks B2–B5), dC+ (blocks B2 and B4–B5), and dV- (block B1) animals. No significant within-group changes over time were found for the dC- controls. Finally, bioacoustic properties—namely call duration and principal frequency (F_0_)—did not differ between dV+ and dC+ animals (S6G and S6H Fig). Due to the low number of vocalizations emitted by dV- and dC- animals (median of 3 calls per block), their acoustic properties were excluded from further statistical analysis.
(TIFF)
