# Supplementary figures and images for: Altered stress and fear responses in the VPA rat model of autism: Behavioral dissociation across tactile, nociceptive, and social contexts
Source: PLoS One. 2026 Jul 17;21(7):e0353839. doi: 10.1371/journal.pone.0353839 (PMC13378972; doi:10.1371/journal.pone.0353839)

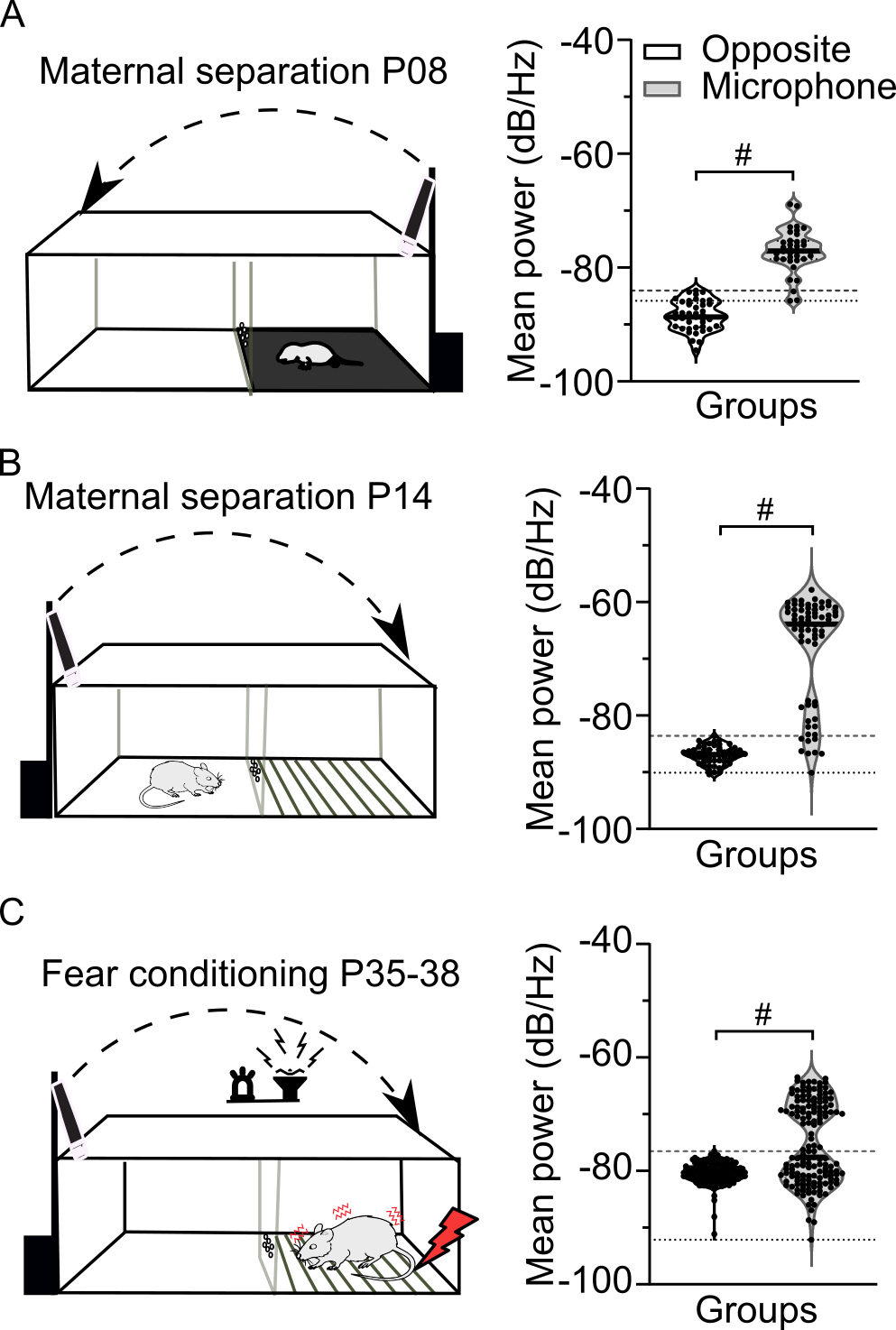

Supplement: S1 Table — Offspring from multiple litters were distributed across experimental conditions to minimize potential confounding effects of litter identity. Within each experimental paradigm, independent litters were assigned to the CTL and VPA groups, such that no litter contributed animals to both treatment conditions. (TIFF) [file pone.0353839.s001.tiff]

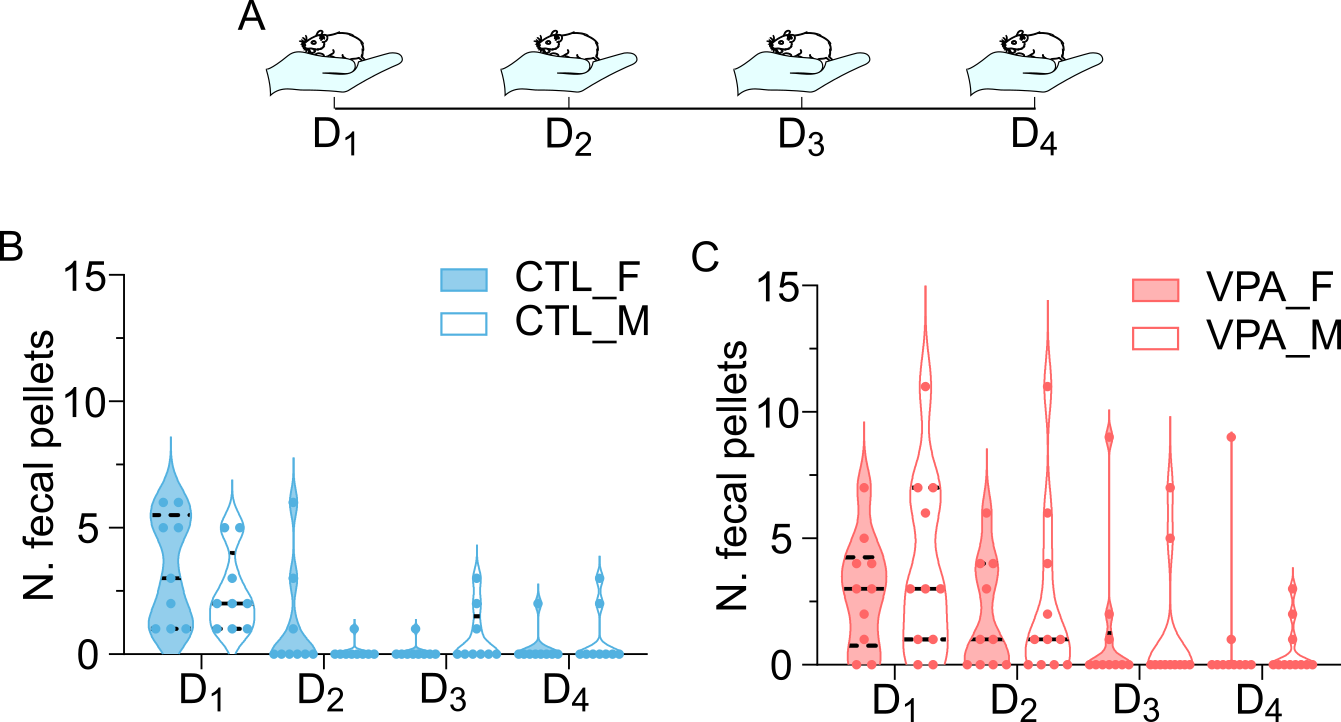

Supplement: S1 Fig — Vocalizations produced during the emotional contagion paradigm were assigned to DEM or OBS animals based on ultrasonic vocalization (USV) power intensity. Thresholds were established experimentally (A–C) by comparing the power of USVs recorded with a microphone positioned either in the same chamber as the vocalizing animal or in the opposite chamber. (A–B) Mean USV power during maternal separation at postnatal days P08 and P14 (#p < 0.0001). (C) Mean USV power during fear conditioning at P35–P38 (#p < 0.0001). Mann–Whitney test. Panel A: nUSV_opposite = 41; nUSV_microphone = 35 (N = 1). Panel B: nUSV_opposite = 55; nUSV_microphone = 69 (N = 1). Panel C: nUSV_opposite = 153; nUSV_microphone = 153 (N = 6). Vocalizations with power > −76 dB were classified as originating from animals located in the microphone chamber (DEM chamber), whereas vocalizations <−85 dB were classified as originating from animals in the opposite chamber (OBS chamber). Vocalizations with power values between −76 and −85 dB were excluded from analyses to minimize ambiguity in chamber assignment. Calls within this interval represented 9.8% of all vocalizations emitted during the emotional contagion experiment (2086/21388 calls), corresponding to 8.4% of CTL calls and 11.2% of VPA calls. (TIFF) [file pone.0353839.s002.tiff]

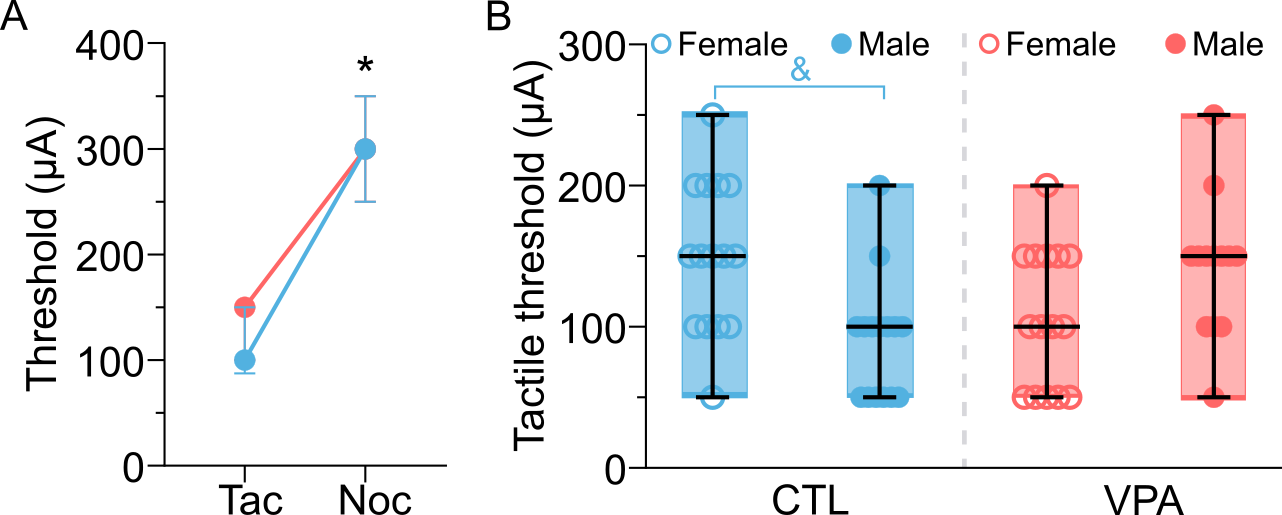

Supplement: S2 Fig — (A) Schematic illustration of the touch sensitivity experimental design. (B) Number of fecal pellets released on each experimental day by female and male CTL animals (p > 0.050). (C) Number of fecal pellets released on each experimental day by female and male VPA-treated animals (p > 0.050). Mann–Whitney test, CTL: n = 18 (9 females, 9 males); VPA: n = 21 (10 females, 11 males). (TIFF) [file pone.0353839.s003.tiff]

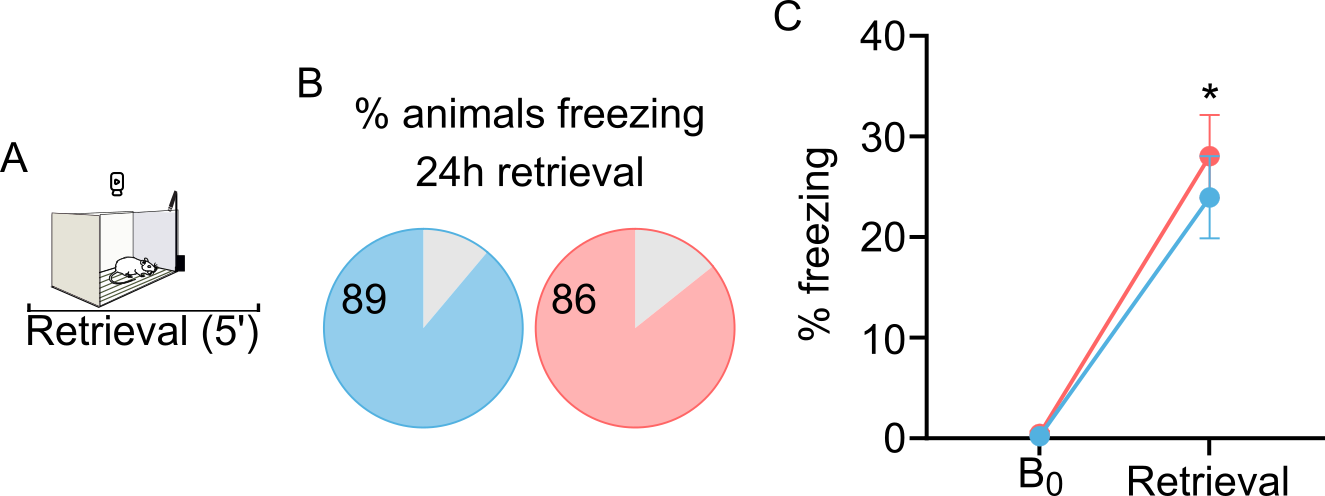

Supplement: S3 Fig — (A) Tactile and nociceptive thresholds (#p > 0.050; *p < 0.001). (B) Tactile thresholds in female and male animals (&p = 0.007). Panel A: paired Student’s t-test. Panel B: Mann–Whitney test. Panel A and B: nCTL = 30 (15 females, 15 males); nVPA = 28 (15 females, 13 males). & indicates significant sex-related differences. (TIFF) [file pone.0353839.s004.tiff]

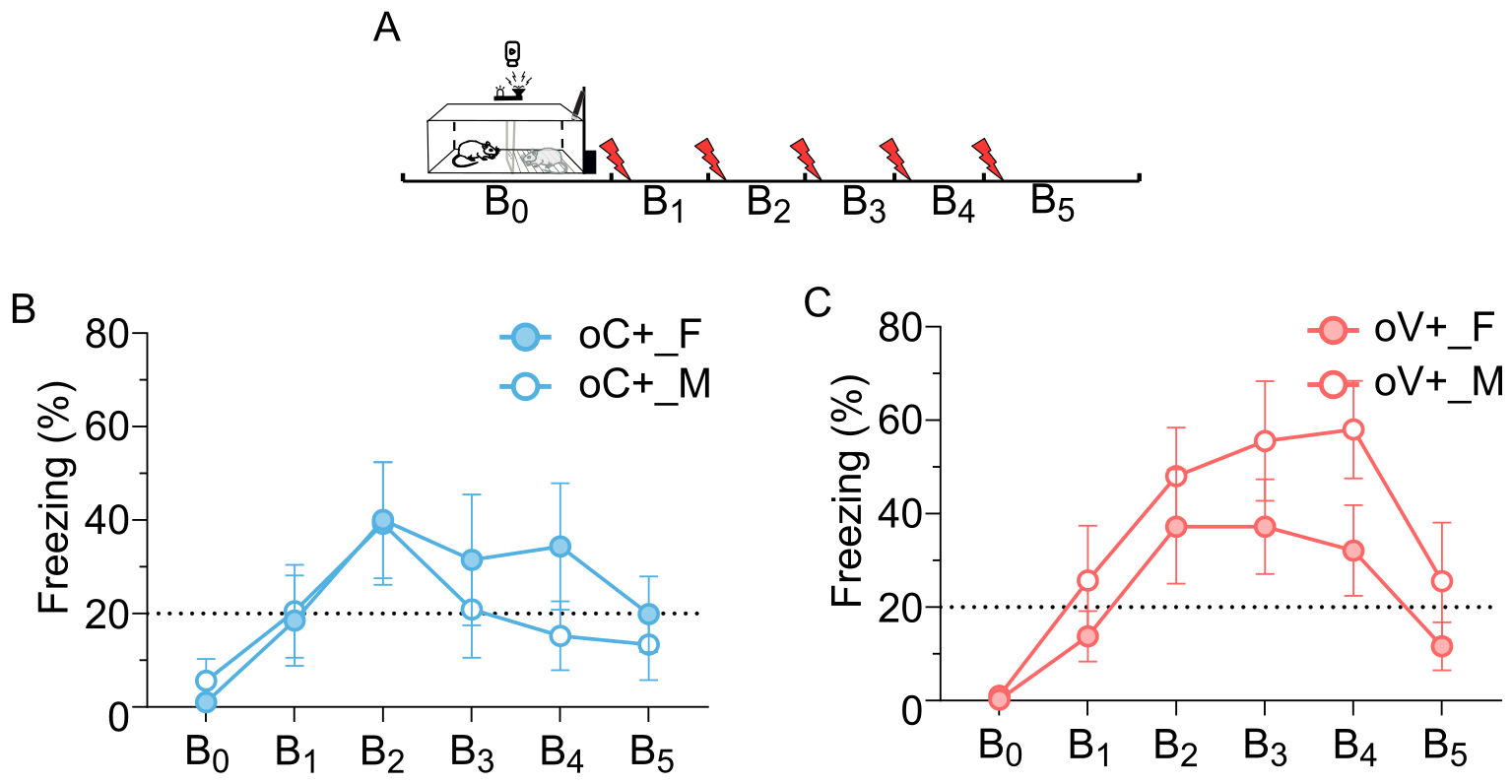

Supplement: S4 Fig — (A) Schematic illustration of the experimental design. Rats were placed in the center of the apparatus facing one of the metal walls and remained in the chamber for 5 min before being returned to their home cage. (B) Percentage of animals displaying freezing behavior during fear conditioning memory retrieval (p > 0.05). (C) Percentage of time spent freezing during the fear conditioning baseline (B0) and memory retrieval session (p < 0.001). Panel B: Fisher’s exact test. Panel C: Student’s t-test and paired Student’s t-test, CTL: n = 27 (13 females, 14 males); VPA: n = 28 (14 females, 14 males). * indicates significant within-group differences. (TIFF) [file pone.0353839.s005.tiff]

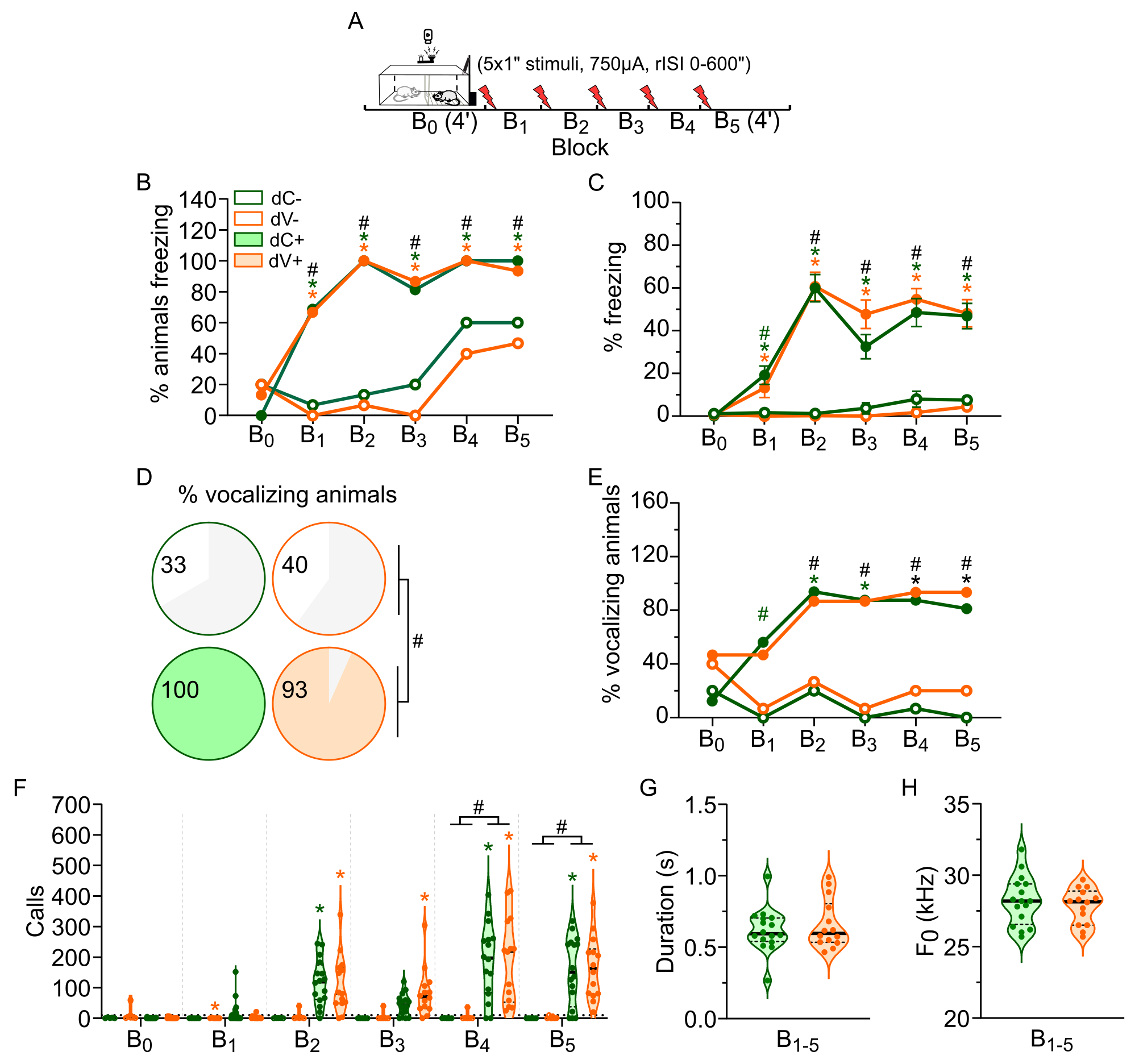

Supplement: S5 Fig — (A) Schematic illustration of the emotional contagion experimental design. (B) Percentage of time spent freezing in each experimental block by female and male CTL animals. (C) Percentage of time spent freezing in each experimental block by female and male VPA-treated animals. The dotted line indicates the 20% reference value. Repeated-measures two-way ANOVA, NoC + : n = 16 (8 females, 8 males); NoV + : n = 15 (8 females, 7 males). (TIFF) [file pone.0353839.s006.tiff]

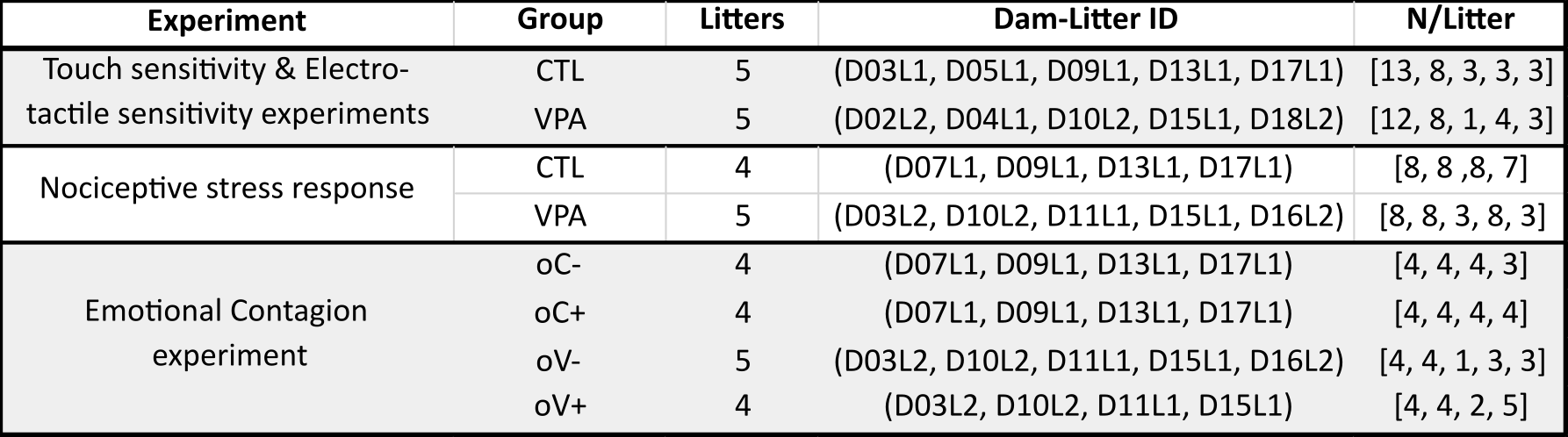

Supplement: S6 Fig — (A) Schematic illustration of the emotional contagion experimental design. Following a 4-min period of free exploration, DEM animals received five electrical shock stimuli. After the final stimulus, animals remained in the chamber for an additional 4 min of free exploration. (B) Percentage of animals displaying freezing behavior in each experimental block (CTL: #p ≤ 0.011; VPA: #p ≤ 0.021; dC + : *p < 0.001; dV + : *p ≤ 0.012). (C) Percentage of time spent freezing by animals in each experimental block (CTL: #p ≤ 0.008; VPA: #p < 0.001; dC + : *p < 0.001; dV + : *p ≤ 0.022). (D) Percentage of animals emitting USVs across the experiment (B0–B5) (CTL: #p < 0.001; VPA: #p ≤ 0.011). (E) Percentage of animals emitting USVs in each experimental block (CTL: #p ≤ 0.002; VPA: #p ≤ 0.006; dC + : *p ≤ 0.042; dV + : *p ≤ 0.027). (F) Number of USVs emitted in each experimental block (CTL: #p ≤ 0.017; VPA: #p ≤ 0.024; dV − : *p = 0.017; dC + : *p ≤ 0.001; dV + : *p ≤ 0.042). (G) Duration of USVs emitted during blocks B1–B5 (p > 0.050). (H) Principal frequency of USVs emitted during blocks B1–B5 (p > 0.050). Panels B, D, and E: Fisher’s exact test. Panel C: repeated-measures two-way ANOVA. Panel F: Friedman test. Panels G–H: Mann–Whitney test. Panels B–E: ndC− = 15 (7 females, 8 males); ndV− = 15 (7 females, 8 males); ndC+ = 16 (8 females, 8 males); ndV+ = 15 (8 females, 7 males). Panel F: noC− = 5 (2 females, 3 males); noV− = 6 (2 females, 4 males); noC+ = 16 (8 females, 8 males); noV+ = 14 (8 females, 6 males). Panels G–H: noC+ = 16 (8 females, 8 males); noV+ = 14 (8 females, 6 males). # indicates significant differences between Shock (+) and non-Shock (−) groups; * indicates significant within-group differences compared with block B0. Because the behavioral responses of observer (OBS) rats are expected to depend on the behavior of demonstrator (DEM) rats, we quantified freezing behavior and ultrasonic vocalizations (USVs) emitted by DEM animals during the emotional contagion pa [file pone.0353839.s007.tiff]
