# Supplementary material for: Altered stress and fear responses in the VPA rat model of autism: Behavioral dissociation across tactile, nociceptive, and social contexts
Source: PLoS One. 2026 Jul 17;21(7):e0353839. doi: 10.1371/journal.pone.0353839 (PMC13378972; doi:10.1371/journal.pone.0353839)
Supplement: Attachment — Submitted filename: Response to Reviewers.docx [file pone.0353839.s010.docx]

**Answers to the Reviewers’ Comments**

Dear Dr. Saka - Academic Editor and the Editorial Board of PLOS ONE,

Thank you for the opportunity to submit our revised manuscript, titled "Altered Stress and Fear Responses in the VPA Rat Model of Autism: Behavioral Dissociation Across Tactile, Nociceptive, and Social Contexts." We would like to express our sincere gratitude to the reviewers for their time, dedication, and highly constructive feedback during this evaluation process. We believe the quality and clarity of the paper have significantly improved with the revisions as we have meticulously considered and addressed each of the commentaries and suggestions. Below, we provide a comprehensive, point-by-point response detailing the modifications implemented, which we believe have significantly strengthened the final manuscript.

**Reviewer #2:** The manuscript looks in great shape now. Well done on this important and needed work. I think it will make a valuable contribution to the field.

R. We thank the reviewer for the comments.

**Reviewer #3:** The article is very methodically explained. However I have a few suggestions

L99-P21 the acronym P to be expanded

R: We thank the reviewer for the comment. We clarified the acronym in the Manuscript as follows “... postnatal day 21 (i.e. P21).” In **L99**.

L165 wav" Format to be replaced by WAV~

R: We thank the reviewer for the comment. We changed it to “WAV” in all places it appeared in the Manuscript. In **L170**.

L316 "significantly prevalence" to be corrected to significant prevalence

R: R: We thank the reviewer for the comment. We revised the Manuscript text.

Originally: “During the electro-tactile sensitivity test (Fig 2A), the VPA group showed a significant prevalence of animals releasing fecal pellets as compared to the CTL group (Fig 2B).”)

Now reads: “During the electro-tactile sensitivity test (Fig 2A), a larger proportion of VPA-treated rats released fecal pellets compared with CTL rats (Fig 2B), indicating increased stress susceptibility.”In **L323**.

L399 VPA treated animals displayed a bimodal distribution of mean call duration in contrast to unimodal pattern " I feel it needs some clarification of this line.

R: We thank the reviewer for the comment. We clarified this point in the Manuscript by adding the following explanation:

“ *vocalizations emitted by VPA-treated rats displayed a bimodal distribution of call duration, characterized by clusters of shorter (~500 ms) and longer (~800 ms) calls, whereas CTL rats exhibited a more unimodal distribution centered around ~600 ms.”* **L405**.

**Reviewer #4: PLOS ONE PONE-D-25-43300R1**
Reviewer: NN
REVIEWER COMMENTS of: Altered Stress and Fear Responses in the VPA Rat Model of Autism: Behavioral Dissociation Across Tactile, Nociceptive, and Social Contexts
The submitted material provides a comprehensive overview of stroke rehabilitation and demonstrates a strong emphasis on clinically relevant concepts, particularly the role of neuroplasticity and multidisciplinary management. However, several aspects could be improved to enhance clarity and academic quality. In terms of content, certain concepts, especially those related to neuroplasticity and the rationale behind specific rehabilitation interventions, would benefit from clearer explanation and more explicit linkage to clinical practice. Additionally, the use of terminology and formatting is somewhat inconsistent throughout the document, and standardization in accordance with established medical guidelines is recommended. The inclusion of up-to-date references would further strengthen the scientific credibility of the material, particularly for statements regarding recovery timelines and therapeutic effectiveness.

Second, some sections, particularly the description of behavioral paradigms and results, are dense and may benefit from clearer structuring.

R: The opening comments of the reviewer are not related to our Manuscript. Our Manuscript has not investigated “... stroke rehabilitation…”.

**1. Absence of hearing assessment critically undermines USV-based conclusions**
The authors themselves acknowledge the lack of auditory assessment as a limitation, but this understates the problem. A significant portion of the results, particularly the emotional contagion findings, depends on the assumption that VPA and CTL observer animals perceived the demonstrator's vocalizations equally. VPA-exposed rats are known to exhibit altered auditory processing and brainstem auditory evoked potential abnormalities (see Gandal et al., 2010; Mehta & Bhatt, 2013). Without audiometric screening, it is impossible to rule out that differences in observer freezing or vocalization patterns between oV+ and oC+ animals reflect differences in auditory sensitivity rather than emotional processing. The authors must either provide hearing data or substantially qualify their emotional contagion conclusions to acknowledge this as a confound, not merely a limitation.

R: We thank the reviewer for this important observation. We agree that auditory function represents a potential confounding factor when interpreting behaviors dependent on acoustic communication, particularly in the emotional contagion paradigm. Direct audiometric or auditory brainstem response measurements were not performed in the present study, and therefore we cannot fully exclude the possibility that subtle alterations in auditory processing contributed to the observed behavioral differences between VPA-treated and control animals. Accordingly, we have revised the Discussion to more explicitly acknowledge this point as a potential confound when interpreting emotional contagion-related behaviors (**L698**).

Previous electrophysiological work from our group (Anomal et al., 2015) suggests that cortical responsiveness to ultrasonic-frequency stimuli remains largely preserved in the VPA rat model. Using high-density recordings in primary auditory cortex (A1), we observed that 36% of recording sites in VPA-treated rats responded to high-frequency (10–50 kHz), low-intensity (<50 dB) stimuli, compared to 40% in control animals, indicating only modest differences in cortical responsiveness to ultrasonic sounds. We therefore interpret our findings cautiously: while existing evidence suggests that gross sensitivity to high-frequency auditory stimuli is relatively preserved in VPA-treated rats, we cannot exclude the possibility that more subtle alterations in auditory perception, salience attribution, or acoustic processing contributed to the behavioral effects observed in the emotional contagion paradigm.

Regarding the study by Daniel Gandal, we note that the experiments were conducted in mice rather than rats, and used low-frequency auditory brainstem response paradigms (6–9 kHz tones), which differ substantially from the ultrasonic distress vocalizations analyzed in the present study. While these findings support the possibility of altered auditory processing in VPA models, species and stimulus differences limit direct extrapolation to the present experimental context. Concerning the citation to Mehta & Bhatt (2013), we were unable to identify a publication by these authors directly related to auditory processing or emotional contagion in the VPA model.

**2. The vocalization chamber-attribution method is insufficiently validated for the main text**
The power-threshold method used to assign USVs to either the demonstrator or observer chamber (>−76 dB = DEM; <−85 dB = OBS) is described only in a supplementary figure derived from a maternal separation pilot. Several issues remain unaddressed: (a) The pilot used neonatal animals at P08 and P14, whereas the emotional contagion experiment was conducted at P41: body size, lung capacity, and call amplitude change substantially across development, making cross-age threshold transfer questionable. (b) There is an unresolved gap between −76 dB and −85 dB where calls cannot be reliably attributed. What proportion of total calls fell in this ambiguous range, and how were they handled? (c) Was any cross-validation performed within the actual emotional contagion dataset? This method is central enough to the paper's main findings that it requires a more rigorous validation and should be described transparently in the Methods section, not relegated to supplementary material.

R: We thank the reviewer for this important methodological observation. Because chamber attribution of ultrasonic vocalizations is central to the interpretation of the emotional contagion experiments, we agree that the procedure required clearer justification and more transparent description in the main text. To address this concern, we revised the Methods section (paragraph starting at **L208**) to include: (1) the rationale underlying the power-threshold attribution approach; (2) the empirical basis used to define the intensity cut-offs; and (3) the handling of calls falling within the ambiguous intensity range. Supplementary Figure S1 was also simplified and now serves as supporting validation material rather than the primary methodological description.

Regarding developmental differences in call amplitude (comment *a*), we agree that body size and lung maturation influence USV intensity across development. To address this issue, Figure S1 includes recordings not only from neonatal maternal separation paradigms (P08 and P14), but also from juvenile fear conditioning experiments (P35–38), which more closely approximate the age and emotional context of the emotional contagion experiments (P41). Across all conditions, calls recorded from the same chamber as the emitter consistently exhibited substantially higher intensities than calls recorded from the opposite chamber, supporting the use of conservative intensity thresholds for chamber attribution. Importantly, the thresholds were intentionally selected to minimize misclassification rather than maximize call inclusion. Vocalizations above −76 dB were classified as demonstrator-derived, whereas vocalizations below −85 dB were classified as observer-derived. Calls within the intermediate range (−76 to −85 dB), which could not be reliably assigned to either chamber, were excluded from analysis.

Regarding the ambiguous range (comment *b*), calls between −76 and −85 dB represented 9.8% of all vocalizations recorded during the emotional contagion experiments (2086/21388 calls; CTL: 8.4%; VPA: 11.2%). Consequently, 91.2% of all calls remained available for analysis after exclusion of potentially ambiguous signals. This exclusion procedure was adopted specifically to reduce chamber-attribution uncertainty. Regarding cross-validation within the emotional contagion dataset (comment *c*), we acknowledge that no direct ground-truth validation of emitter identity was performed during these sessions. We now explicitly recognize this as a methodological limitation in the revised manuscript (**L698**). Nevertheless, the use of conservative cut-offs together with exclusion of intermediate-intensity calls was intended to minimize assignment ambiguity and reduce the likelihood of systematic misclassification.

Finally, all methodological details regarding chamber attribution thresholds and exclusion criteria have now been incorporated into the main Methods section (paragraph starting at **L208**), rather than being described exclusively in the Supplementary Materials.

Included in the **Discussion section (L648)**:

*“Although we cannot entirely exclude the possibility that subtle differences in auditory sensitivity contribute to the observed behavioral variations between VPA-treated and control animals, our previous research indicates a difference of less than 5% in cortical sensitivity for high-frequency sounds. Specifically, high-density cortical recordings showed that 36% of A1 sites in VPA-treated rats were responsive to high-frequency (10–50 kHz), low-intensity (<50 dB) stimuli, compared to 40% in control animals (68)”*.

**3. Litter effects are inadequately controlled**
The authors argue that distributing offspring across groups constitutes sufficient litter-effect control. While this randomization strategy reduces confounding, it does not eliminate it, and the claim that statistical adjustment was therefore "unnecessary" is not well-supported. The number of litters is relatively small (8 VPA-producing litters, 6 CTL-producing litters), and some females contributed two litters. Given that litter is a recognized source of non-independence in rodent behavioral studies (Lazic & Essioux, 2013), a sensitivity analysis, such as a mixed model with litter as a random effect, or at minimum a report of ICC values, should be provided to demonstrate that litter-level clustering did not inflate false-positive rates.

We thank the reviewer for raising this important point. We agree that balanced allocation of offspring across experimental groups reduces, but does not eliminate, potential litter-related non-independence. Accordingly, in the revised analyses we explicitly evaluated litter-level clustering using linear mixed-effects models including litter as a random effect, consistent with the recommendations of Lazic & Essioux.

As part of the experimental design, offspring from multiple litters were distributed across experimental conditions to minimize potential confounding between treatment and litter identity. In each experiment, independent litters were used for the CTL and VPA groups, such that no litter contributed animals to both treatment conditions within the same experimental paradigm. Detailed litter composition for each experiment has now been included in the **Supplementary Materials** (**Table S1**). Overall, the study included animals derived from 14 independent litters (6 CTL-derived and 8 VPA-derived litters). Some litters contributed offspring to more than one behavioral experiment.

**Table S1:** **Summary table of all animals and litters used in the experiments.** Offspring from multiple litters were distributed across experimental conditions to minimize potential confounding between treatment and litter identity. In each experiment, independent litters were used for the CTL and VPA groups, such that no litter contributed animals to both treatment conditions within the same experimental paradigm.

| **Experiment** | **Group** | **Litters** | **Dam-Litter ID** | **N/Litter** |
| --- | --- | --- | --- | --- |
| Touch sensitivity & Electro-tactile sensitivity experiments | CTL | 5 | (D03L1, D05L1, D09L1, D13L1, D17L1) | [13, 8, 3, 3, 3] |
|  | VPA | 5 | (D02L2, D04L1, D10L2, D15L1, D18L2) | [12, 8, 1, 4, 3] |
| Nociceptive stress response & Fear Conditioning experiments | CTL | 4 | (D07L1, D09L1, D13L1, D17L1) | [8, 8 ,8, 7] |
|  | VPA | 5 | (D03L2, D10L2, D11L1, D15L1, D16L2) | [8, 8, 3, 8, 3] |
| Emotional Contagion experiment | oC- | 4 | (D07L1, D09L1, D13L1, D17L1) | [4, 4, 4, 3] |
|  | oC+ | 4 | (D07L1, D09L1, D13L1, D17L1) | [4, 4, 4, 4] |
|  | oV- | 5 | (D03L2, D10L2, D11L1, D15L1, D16L2) | [4, 4, 1, 3, 3] |
|  | oV+ | 4 | (D03L2, D10L2, D11L1, D15L1) | [4, 4, 2, 5] |

To directly address the reviewer’s concern, we performed complementary mixed-effects sensitivity analyses on the emotional contagion dataset.

First, we analyzed the individual-animal repeated-measures data using a linear mixed-effects model taking into account litter as random effect. The selected model included Group, Treatment, and Treatment × Block as fixed effects, with random intercepts for litter and individual animals nested within litter. This analysis revealed a highly significant Treatment × Block interaction (F(5,352) = 10.729, p = 1.30 × 10^−9^), indicating that freezing dynamics across experimental blocks differed as a function of treatment condition. Importantly, the estimated litter intraclass correlation coefficient was extremely small: ICC_Litter = 0.0069 indicating that less than 1% of the total variance was attributable to litter of origin, i.e., animals from the same litter behaved almost independently with respect to the analyzed outcome. In contrast, repeated measures within individual animals accounted for a substantially larger proportion of variance, supporting the conclusion that the observed behavioral effects primarily reflected individual behavioral dynamics rather than litter-specific clustering. Second, as a conservative sensitivity analysis, we averaged freezing values within litters and reanalyzed the data using litter-level observations. Despite the substantial reduction in statistical power resulting from litter averaging, the Treatment × Block interaction remained statistically significant (F(5,88) = 3.078, p = 0.013). Moreover, the estimated litter ICC in this conservative analysis was effectively zero: ICC_Litter≈0. *(*) Six alternative linear mixed-effects models differing in their fixed- and random-effects structures were compared. The final model was selected based on the best balance between model fit and parsimony using the Bayesian Information Criterion (BIC).*

Together, these complementary analyses indicate that the principal findings were robust to litter-level aggregation and were not driven by pseudo-replication or litter-specific clustering effects. We additionally calculated ICCs for other principal behavioral and vocalization measures across the study. The estimated ICCs were: Call duration: 0.040; Fundamental frequency: 0.146; Number of USVs: 0.018. These values indicate that litter contributed only a small-to-moderate proportion of total variance across measures. Importantly, the major experimental effects remained statistically consistent after inclusion of litter as a random factor. We have incorporated clarifications into the revised manuscript as follows:

**Added to Material and Methods - Animals (L111)**

*“Offspring from the same litter was balanced distributed across the groups and mixed-effects analyses were performed to explicitly account for litter-level clustering.”*

**Added to the Material and Methods - Statistics section (L277)**

*“To evaluate potential litter effects in the social stress response experiment, we applied linear mixed-effects models including litter as a random effect and additionally performed a sensitivity analysis in which outcomes were averaged within each litter and statistical comparisons were repeated using litter means as independent observations.”*

**Added to the Results section (L497)**

*“Linear mixed-effects analyses including litter as a random effect were performed to account for potential non-independence among animals from the same litter. This analysis revealed a highly significant Treatment × Block interaction (F(5,352) = 10.729, p = 1.30 × 10−9), indicating that freezing dynamics across experimental blocks differed as a function of treatment condition. Importantly, the estimated litter intraclass correlation coefficient was extremely small: ICC_Litter = 0.0069 indicating that less than 1% of the total variance was attributable to litter of origin, i.e., animals from the same litter behaved almost independently with respect to the analyzed outcome. In addition, as a conservative sensitivity analysis, we averaged freezing values within litters and reanalyzed the data using litter-level observations. Despite the substantial reduction in statistical power resulting from litter averaging, the Treatment × Block interaction remained statistically significant (F(5,88) = 3.078, p = 0.013). Moreover, the estimated litter ICC in this conservative analysis was effectively zero, demonstrating that the reported effects were robust to litter-based clustering and not attributable to pseudo-replication.”*

**4. Interpretive overreach in the fear conditioning dissociation findings**
The authors present the absence of correlation between freezing, defecation, and vocalization in VPA rats as evidence of "disrupted coordination of fear responses" and "impaired integration of emotional processing." This is an interesting hypothesis, but the interpretation rests on null results from correlation analyses conducted in **subgroups** that are notably **small** (**9 VPA animals for the freezing/USV correlation**). The statistical power to detect correlations of moderate effect size (r ~0.5) in a sample of 9 is approximately 30–40%, meaning failure to detect a correlation is highly uninformative. The authors should either **(a) calculate and report post-hoc power for these correlation analyses, or (b) substantially temper their interpretive language to reflect that absence of evidence is not evidence of disrupted integration**, particularly in underpowered subgroup analyses.

R: We thank the reviewer for this important observation regarding the interpretation of the correlation analyses.

Following the reviewer’s suggestion, we reanalyzed the correlations using a broader and more inclusive criterion for responsive animals. Specifically, animals were considered responsive if they exhibited at least one freezing episode (≥ 3 s), regardless of whether they emitted ultrasonic vocalizations or released fecal pellets. This approach substantially increased the number of animals included in the analyses and avoided restricting the correlations to small behavioral subgroups.

In the fear conditioning experiment, only one CTL animal failed to display freezing behavior, whereas all animals in the oC+ and oV+ groups exhibited freezing, in the analyzed blocks (B1-B5). Under these conditions, the positive association between freezing behavior and ultrasonic vocalizations remained evident in both CTL and VPA-treated animals.

Importantly, the relationship between freezing behavior and fecal pellet release remained significantly altered in VPA animals even in the full mixed-model analysis. Specifically, we observed a significant Group × Freezing interaction (F(1,51)=5.05, p=0.029), demonstrating that the coupling between defensive freezing and autonomic emotional output differed between CTL and VPA-treated animals. Whereas CTL animals showed a robust negative association between freezing and defecation, this relationship was markedly attenuated in VPA-treated animals. Because this effect was detected using the full dataset (27 CTL and 29 VPA animals), rather than small subgroups, and remained significant after accounting for litter-related non-independence, we believe the data support a more circumscribed interpretation that prenatal VPA exposure alters the coordination between specific components of fear responding, particularly between behavioral freezing and autonomic visceral output.

In addition, analyses performed during the emotional contagion paradigm revealed no significant associations between freezing behavior and USV production in either CTL or VPA-treated animals, as well as no significant Group or Treatment effects. These findings suggest that freezing–USV coupling is not uniformly present across emotional paradigms, reinforcing the interpretation that the altered freezing–defecation relationship observed during fear conditioning reflects a selective disruption of specific defensive-response coordination mechanisms rather than a generalized breakdown of emotional processing.

Accordingly, we revised the manuscript to distinguish between:
 (i) unsupported claims regarding disrupted freezing–USV coupling, and
 (ii) statistically supported evidence for altered freezing–defecation integration in VPA-treated animals.

**Revisions implemented:**

(1) In the **Abstract**, we modified. Now reads:

**L31:** *“During fear conditioning, VPA rats demonstrated a delayed onset but subsequent enhancement of freezing responses, alongside disrupted temporal coordination between freezing and defecation behaviors. 22-kHz USVs positively correlated with freezing across both groups.”*

**L38:** *“These findings indicate that VPA-treated rats display heightened stress reactivity, habituation deficit and suggest disrupted coordination of fear responses, supporting the VPA model as a relevant tool for investigating the neurobiological basis of stress vulnerability and social dysfunction in ASD.”*

(2) **In the Results section**, we included:

**L418:** *“To further evaluate this relationship, we analyzed the complete dataset (27 CTL and 29 VPA animals) using a linear mixed-effects model including group and sex as fixed effects and litter as a random intercept to account for litter-related non-independence. The analysis confirmed a significant association between freezing and USV emission (F(1,51) = 23.92, p < 0.001), with no significant Group × Freezing interaction (F(1,51) = 0.79, p > 0.05), which indicates that the correlation between the variables do not depend on the treatment. Consistent with this interpretation, correlation showed that although significant positive correlations were detected in CTL (⍴ = 0.51, p = 0.007) and VPA groups (⍴ = 0.45, p = 0.014), there was no difference between them. In contrast, freezing behavior and fecal pellet release showed a significant negative association (F(1,51) = 10.63, p = 0.002) that was primarily driven by CTL animals (CTL: ⍴ = −0.502, p = 0.008; VPA: ⍴ = −0.038, p = 0.846). Moreover, a significant Group × Freezing interaction was observed (F(1,51) = 5.05, p = 0.029), indicating that the relationship between freezing behavior and defecation differed between CTL and VPA-treated animals. Specifically, freezing behavior was negatively associated with pellet release in CTL animals, whereas this relationship was markedly attenuated in VPA-treated animals.These observations further support our previous results.”*

(3) We refined and adapted the **Discussion section** accordingly to incorporate the Reviewer’s suggestions.

(4) **In the Conclusion section** **(L709)**now reads: “Our findings also provide evidence that animals prenatally exposed to VPA have altered coordination between freezing response and autonomic visceral control.”

**5. Inconsistent block-level analysis across paradigms**
The fear conditioning and emotional contagion paradigms use block-by-block analysis (B0–B5), but the criteria for what constitutes a "responsive" animal differ across measures and are not consistently applied. For freezing, responsiveness is defined as displaying any freezing during the experiment; for vocalizations, it is defined as emitting at least one USV. These thresholds are reasonable individually, but the resulting subgroup sizes shift considerably across analyses within the same experiment (e.g., N = 27 for freezing vs. N = 22 for USV in CTL fear conditioning).
Overall, the content is informative and relevant, and with refinement in structure, clarity, and evidence support, it has strong potential to serve as an effective educational resource.

R: We thank the reviewer for this important observation. Because freezing behavior, ultrasonic vocalizations, and defecation represent partially dissociable components of defensive and emotional responding, differences in the number of animals expressing each behavior were expected and reflect genuine behavioral variability rather than inconsistent analytical procedures. Our objective was not to compare subgroup sizes across behavioral domains, but rather to characterize each behavioral output independently and subsequently evaluate the relationships among them. Correlation analyses were therefore performed to determine whether these behavioral measures represented redundant manifestations of a common emotional state or, alternatively, partially independent dimensions of the stress response.

Nevertheless, to address the reviewer’s concern regarding statistical power, we revised the correlation analyses using a more inclusive criterion for responsive animals. Specifically, animals were included whenever they exhibited at least one freezing episode (≥ 3 s), independently of whether they emitted ultrasonic vocalizations or released fecal pellets. This approach substantially increased the sample size in the fear conditioning experiment. However, its impact on the emotional contagion dataset was minimal because virtually all animals exhibited at least one freezing episode. Only two animals from the oC− group failed to meet this criterion and were excluded, whereas all animals from the oC+, oV−, and oV+ groups were included in the correlation analyses. Thus, the revised emotional contagion analyses were conducted using nearly the entire experimental population.

To improve clarity, we have revised the Methods section to explicitly describe the responsiveness criteria used for each behavioral measure in both the fear conditioning and emotional contagion experiments (**L262**): In the behavioral tests, animals were considered “responsive” based on minimal engagement criteria specific to each paradigm. When evaluating freezing behavior, an animal was considered responsive if it exhibited at least one freezing episode (≥ 3 s) during the experimental session. When evaluating ultrasonic vocalizations, responsiveness was defined as the emission of at least one ultrasonic call. Correlation analyses involving freezing behavior included all animals exhibiting at least one freezing episode, irrespective of ultrasonic vocalization emission or fecal pellet release.

***PS. NOTES*:**

(1) Illustrative images of ultrasonic vocalizations recorded during the fear conditioning experiment were incorporated to Fig. 3. Figure legend was modified accordingly.
