# Supplementary material for: Altered stress and fear responses in the VPA rat model of autism: Behavioral dissociation across tactile, nociceptive, and social contexts
Source: PLoS One. 2026 Jul 17;21(7):e0353839. doi: 10.1371/journal.pone.0353839 (PMC13378972; doi:10.1371/journal.pone.0353839)
Supplement: Peer review history

# Peer review: Decision Letter 0

**DOI:** 10.1371/journal.pone.0353839.r001

**Authors:** Hualin Fu

17 Oct 2025

PONE-D-25-43300

Altered Stress and Fear Responses in the VPA Rat Model of Autism: Behavioral Dissociation Across Tactile, Nociceptive, and Social Contexts

PLOS ONE

Dear Dr. Hashiguchi,

Thank you for submitting your manuscript to PLOS ONE. After careful consideration, we have decided that your manuscript does not meet our criteria for publication and must therefore be rejected.

I am sorry that we cannot be more positive on this occasion, but hope that you appreciate the reasons for this decision.

Kind regards,

Hualin Fu

Academic Editor

PLOS ONE

[Note: HTML markup is below. Please do not edit.]

Reviewers' comments:

Reviewer's Responses to Questions

**Comments to the Author**

1. Is the manuscript technically sound, and do the data support the conclusions?

The manuscript must describe a technically sound piece of scientific research with data that supports the conclusions. Experiments must have been conducted rigorously, with appropriate controls, replication, and sample sizes. The conclusions must be drawn appropriately based on the data presented.

Reviewer #1: No

**********

2. Has the statistical analysis been performed appropriately and rigorously?

Reviewer #1: N/A

**********

3. Have the authors made all data underlying the findings in their manuscript fully available?

The PLOS Data policy requires authors to make all data underlying the findings described in their manuscript fully available without restriction, with rare exception (please refer to the Data Availability Statement in the manuscript PDF file). The data should be provided as part of the manuscript or its supporting information, or deposited to a public repository. For example, in addition to summary statistics, the data points behind means, medians and variance measures should be available. If there are restrictions on publicly sharing data—e.g. participant privacy or use of data from a third party—those must be specified.

Reviewer #1: Yes

**********

4. Is the manuscript presented in an intelligible fashion and written in standard English?

PLOS ONE does not copyedit accepted manuscripts, so the language in submitted articles must be clear, correct, and unambiguous. Any typographical or grammatical errors should be corrected at revision, so please note any specific errors here.

Reviewer #1: Yes

**********

5. Review Comments to the Author

Please use the space provided to explain your answers to the questions above. You may also include additional comments for the author, including concerns about dual publication, research ethics, or publication ethics. (Please upload your review as an attachment if it exceeds 20,000 characters)

Reviewer #1: This manuscript reports stress and sensory-related behaviours in rats exposed to valproic acid (VPA), claiming relevance to autistic people. The authors measure freezing, tactile and nociceptive thresholds, fecal pellet counts, and ultrasonic vocalisations, and interpret these as indicators of autism-relevant traits.

Major Concerns

1. Fundamentally Flawed Conceptual and Ethical Basis

This research is deeply and fundamentally flawed for two reasons:

a. The study repeatedly treats autistic people as having a disorder, rather than recognising increasing volumes of research suggesting that autism is a naturally occurring neurotype. This deficit-framed perspective is outdated and misrepresents autistic lived experience.

b. The study assumes that torturing rats through electric shocks, social stress, and forced handling, can meaningfully inform understanding of autistic people. It cannot. Autistic people have not requested this research, and the procedures offer no translational benefit or insight that could improve their lives. The “nothing about us without us” principle is completely ignored.

These flaws are existential: no matter how well the experiments were conducted or how sophisticated the analyses, the paper’s foundation is scientifically and ethically unsound. The research cannot be justified in any form, and the claimed translational relevance is entirely illusory.

Minor Concerns / Supporting Points

Even if the fundamental ethical and conceptual issues were ignored, the manuscript suffers from numerous additional weaknesses:

• Inconsistent paradigm definitions: The four paradigms described (manipulation, electro-tactile, fear conditioning, emotional contagion) are poorly defined and sometimes conflated, making replication impossible.

• Non-independence of subjects: No statistical adjustment for litter effects.

• Statistical section issues: Numerous tests are listed without mapping to specific analyses, some descriptions are conceptually inaccurate, and there is no mention of pre-registration or a plan for multiple-comparison corrections.

• Cherry-picked results: Subheadings pre-frame positive findings, downplaying null results.

These issues reinforce the conclusion that, even if fixed, the paper would remain irredeemable because of the fundamental flaw in purpose, framing, and ethical justification.

Recommendation

I recommend rejection. This study imposes severe, unnecessary distress on animals while relying on outdated deficit-based models of autism. Its methods, statistical analyses, and behavioural measures are irrelevant to the lives or experiences of autistic people. There is no clear translational benefit, no human consultation, and no defensible rationale for the procedures. The research, in both conception and execution, is entirely inappropriate.

**********

6. PLOS authors have the option to publish the peer review history of their article (what does this mean?). If published, this will include your full peer review and any attached files.

If you choose “no”, your identity will remain anonymous but your review may still be made public.

**Do you want your identity to be public for this peer review?**  For information about this choice, including consent withdrawal, please see our Privacy Policy.

Reviewer #1: No

**********

[NOTE: If reviewer comments were submitted as an attachment file, they will be attached to this email and accessible via the submission site. Please log into your account, locate the manuscript record, and check for the action link "View Attachments". If this link does not appear, there are no attachment files.]

- - - - -

For journal use only: PONEDEC3

# Author comment: Author response to Decision Letter 1

**DOI:** 10.1371/journal.pone.0353839.r002

2 Feb 2026

Dear Reviewer,

We sincerely thank for the time and effort dedicated to evaluating our manuscript. We appreciate the opportunity to clarify the conceptual and ethical framework of our study, particularly in response to the major concern entitled “Fundamentally Flawed Conceptual and Ethical Basis.”

We fully recognize that research involving autism-related topics requires both scien-tific rigor and sensitivity to evolving societal perspectives. Our intention has never been to impose or reinforce deficit-based narratives, but rather to investigate specific neurobiological mechanisms underlying stress and sensory processing using a well-established preclinical model.

The following responses aim to clarify the conceptual scope, ethical compliance, and translational rationale of the study. We respectfully address each of the reviewer’s points — conceptual, ethical, and methodological — providing detailed explanations, relevant literature references, and explicit evidence of adherence to institutional and international standards for animal research.

Our goal is to ensure that the discussion remains within the scientific and methodolog-ical boundaries appropriate for preclinical behavioral neuroscience, while acknowledg-ing the importance of broader debates on neurodiversity and participatory research in human contexts.

***********************************************************************

MAJOR CONCERN: “Fundamentally Flawed Conceptual and Ethical Basis”

(a) “The study treats autistic people as having a disorder, rather than recognising autism as a naturally occurring neurotype.”

Author’s answer: We respectfully disagree with this interpretation. Our study does not aim to define autism as a “disorder” in a sociocultural sense, nor to describe the lived experience of autistic individuals. Rather, it investigates specific neurobiological mechanisms associated with stress and sensory processing in a well-established preclinical model. The terminology Autism Spectrum Disorder (ASD) is employed strictly in accordance with the current diagnostic frameworks — the DSM-5-TR (American Psychiatric Association, 2022) and ICD-11 (World Health Organization, 2022) — which remain the international standards for biomedical and translational research.

Our use of this terminology is therefore descriptive, not ideological. The focus of the work is mechanistic — to examine how prenatal valproic acid exposure alters fear and stress reactivity across different behavioral domains. The findings are interpreted in light of biological processes that may underlie stress vulnerability, a trait frequently documented in autistic populations.

We fully acknowledge the growing movement toward neurodiversity-affirming perspectives and agree that conceptual discussions on autism should evolve with community insights. However, these discussions, while essential at the societal and policy level, are distinct from the methodological focus of preclinical neurobiology, which seeks to isolate variables and mechanisms in controlled experimental settings.

(b) “The study assumes that torturing rats through electric shocks, social stress, and forced handling can meaningfully inform understanding of autistic people… The ‘nothing about us without us’ principle is completely ignored.”

Author’s answer: We must respectfully clarify that no procedures in this study involved torture, cruelty, or unnecessary suffering. All protocols were approved by the Institutional Animal Care and Use Committee of the Federal University of Rio Grande do Norte (UFRN) and conducted in compliance with Brazilian CONCEA guidelines and international standards for animal welfare (NIH Guide for the Care and Use of Laboratory Animals, 2011).

Every effort was made to minimize distress and discomfort, in line with the 3Rs principles (Replacement, Reduction, and Refinement). The brief electro-tactile stimulation used in the fear-conditioning paradigm involved mild, non-injurious intensities (0.4–0.6 mA) comparable to standard learning protocols widely published in high-impact behavioral neuroscience journals. Social-stress and tactile-manipulation procedures followed humane and ethically approved designs, avoiding physical harm or deprivation.

Regarding translational relevance, the study’s aim is mechanistic, not clinical. Animal models cannot replicate the full complexity of autism, but they provide controlled access to specific neurodevelopmental and stress-response pathways that are inaccessible in human experimentation. Preclinical work remains essential for understanding underlying biological processes that may later inform human research — a principle explicitly supported by PLOS ONE’s Animal Research Policy.

Finally, the “nothing about us without us” principle, while deeply relevant for human-subject research and participatory design, is not directly applicable to preclinical studies. Nevertheless, we recognize its ethical spirit and fully support increasing dialogue between the autism community and neuroscience researchers.

MINOR CONCERNS

(a) “Inconsistent paradigm definitions.”

Author’s answer: Each behavioral paradigm is independently and clearly defined in the Methods section:

• Manipulation test — standardized tactile exploration protocol (Bigelow et. al., 2023; Costa et. al., 2012; Hurst et. al., 2010).

• Electro-tactile conditioning — single-trial fear-conditioning paradigm with explicit stimulus parameters (duration, intensity, context).

• Fear conditioning — standard Pavlovian pairing, with full contextual and temporal details (Banerjee et. al., 2014; Maren 2001; Markram et. al., 2008; Lin et. al., 2013; Tovote et. al., 2015; Johansen et. al., 2011).

• Emotional contagion — two-chamber observational paradigm with minor adaptations to Atsak et. al., 2011; Han et. al., 2020; Carrijo et. al., 2019).

Definitions are not conflated; their sequence was designed to assess distinct affective and sensory domains (tactile reactivity, nociceptive learning, social empathy). Replicability is ensured by detailed procedural descriptions and accompanying parameter tables.

(b) “Non-independence of subjects; no statistical adjustment for litter effects.”

Author’s answer: Litter effects were controlled by design, not post hoc. Offspring from each litter were distributed across experimental groups, ensuring that no single litter contributed exclusively to one condition. This randomization strategy is widely accepted as an effective control for litter confounds. As such, statistical adjustment for litter was unnecessary and would have reduced statistical power without improving validity.

(c) “Statistical section issues.”

Author’s answer: All analyses are explicitly mapped to the corresponding tests in the Results section. Parametric assumptions were verified (normality, homogeneity), and appropriate tests were applied (ANOVA, post-hoc Tukey, or non-parametric alternatives as stated). Corrections for multiple comparisons were clearly specified (Holm–Bonferroni), in accordance with PLOS ONE’s Statistical Reporting Guidelines.

No claim in the manuscript depends on uncorrected statistics. The description of procedures follows standard behavioral-neuroscience reporting practices, ensuring transparency and reproducibility.

(d) “Cherry-picked results.”

Author’s answer: This assertion is incorrect. While section subheadings highlight significant findings for readability, null results are also reported and discussed in the text. For example, the emotional-contagion and tactile-manipulation tasks include explicit reporting of non-significant group differences. Figures include means ± SEM for all conditions, and supplementary tables list full test statistics.

Our presentation adheres to PLOS ONE’s data-transparency standards and aims to maintain a balanced discussion of both positive and null outcomes.

CONCLUSION

We respectfully maintain that the manuscript is scientifically sound, methodologically rigorous, and ethically compliant with institutional and international guidelines.

The criticisms raised — while valuable in reflecting broader ethical and conceptual debates about autism — extend beyond the scope of the study’s scientific aims and PLOS ONE’s stated review criteria (validity, rigor, and ethical compliance).

We therefore request that the paper be re-evaluated by reviewers with specific expertise in behavioral neuroscience, stress physiology, or translational models of neurodevelopment, to ensure that its scientific merit is assessed within the appropriate disciplinary framework.

Sincerely,

Debora Hashiguchi, PhD.

# Peer review: Decision Letter 1

**DOI:** 10.1371/journal.pone.0353839.r003

**Authors:** Hualin Fu, Vara Prasad Saka

6 Apr 2026

PONE-D-25-43300R1

Altered Stress and Fear Responses in the VPA Rat Model of Autism: Behavioral Dissociation Across Tactile, Nociceptive, and Social Contexts

PLOS One

Dear Dr. Hashiguchi,

Thank you for submitting your manuscript to PLOS ONE. After careful consideration, we feel that it has merit but does not fully meet PLOS ONE’s publication criteria as it currently stands. Therefore, we invite you to submit a revised version of the manuscript that addresses the points raised during the review process.

Please submit your revised manuscript by May 21 2026 11:59PM. If you will need more time than this to complete your revisions, please reply to this message or contact the journal office at plosone@plos.org. When you're ready to submit your revision, log on to https://www.editorialmanager.com/pone/ and select the 'Submissions Needing Revision' folder to locate your manuscript file.

Please include the following items when submitting your revised manuscript:

- A letter that responds to each point raised by the academic editor and reviewer(s). You should upload this letter as a separate file labeled 'Response to Reviewers'.
- A marked-up copy of your manuscript that highlights changes made to the original version. You should upload this as a separate file labeled 'Revised Manuscript with Track Changes'.
- An unmarked version of your revised paper without tracked changes. You should upload this as a separate file labeled 'Manuscript'.

If you would like to make changes to your financial disclosure, please include your updated statement in your cover letter. Guidelines for resubmitting your figure files are available below the reviewer comments at the end of this letter.

If applicable, we recommend that you deposit your laboratory protocols in protocols.io to enhance the reproducibility of your results. Protocols.io assigns your protocol its own identifier (DOI) so that it can be cited independently in the future. For instructions see: https://journals.plos.org/plosone/s/submission-guidelines#loc-laboratory-protocols. Additionally, PLOS ONE offers an option for publishing peer-reviewed Lab Protocol articles, which describe protocols hosted on protocols.io. Read more information on sharing protocols at https://plos.org/protocols?utm_medium=editorial-email&utm_source=authorletters&utm_campaign=protocols.

As the corresponding author, your ORCID iD is verified in the submission system and will appear in the published article. PLOS supports the use of ORCID, and we encourage all coauthors to register for an ORCID iD and use it as well. Please encourage your coauthors to verify their ORCID iD within the submission system before final acceptance, as unverified ORCID iDs will not appear in the published article. *Only*  the individual author can complete the verification step; PLOS staff *cannot*  verify ORCID iDs on behalf of authors.

We look forward to receiving your revised manuscript.

Kind regards,

Vara Prasad Saka

Academic Editor

PLOS One

Journal Requirements:

When submitting your revision, we need you to address these

additional requirements.

1.Please ensure that your manuscript meets PLOS ONE's style

requirements, including those for file naming. The PLOS ONE style templates can

be found at

https://journals.plos.org/plosone/s/file?id=wjVg/PLOSOne_formatting_sample_main_body.pdf

and

https://journals.plos.org/plosone/s/file?id=ba62/PLOSOne_formatting_sample_title_authors_affiliations.pdf

2. Thank you for stating the following financial disclosure:

“This work was supported by the Coordenação de

Aperfeiçoamento de Pessoal de Nível Superior (CAPES, Brazil;

88882.376056/2019-1 to DH). The funders had no role in study design, data

collection and analysis, decision to publish, or preparation of the manuscript.”

Please state what role the funders took in the study. If the funders had no role, please state:

"The funders had no role in study design, data collection and analysis,

decision to publish, or preparation of the manuscript."

If this statement is not correct you must amend it as

needed.

Please include this amended Role of Funder statement in your

cover letter; we will change the online submission form on your behalf.

3. When completing the data availability statement of the

submission form, you indicated that you will make your data available on

acceptance. We strongly recommend all authors decide on a data sharing plan

before acceptance, as the process can be lengthy and hold up publication

timelines. Please note that, though access restrictions are acceptable now,

your entire data will need to be made freely accessible if your manuscript is

accepted for publication. This policy applies to all data except where public

deposition would breach compliance with the protocol approved by your research

ethics board. If you are unable to adhere to our open data policy, please

kindly revise your statement to explain your reasoning and we will seek the

editor's input on an exemption. Please be assured that, once you have provided

your new statement, the assessment of your exemption will not hold up the peer

review process.

4. We note that you have indicated that there are

restrictions to data sharing for this study. PLOS only allows data to be

available upon request if there are legal or ethical restrictions on sharing

data publicly. For more information on unacceptable data access restrictions,

please see

http://journals.plos.org/plosone/s/data-availability#loc-unacceptable-data-access-restrictions.

Before we proceed with your manuscript, please address the

following prompts:

a) If there are ethical or legal restrictions on sharing a

de-identified data set, please explain them in detail (e.g., data contain

potentially identifying or sensitive patient information, data are owned by a

third-party organization, etc.) and who has imposed them (e.g., a Research

Ethics Committee or Institutional Review Board, etc.). Please also provide

contact information for a data access committee, ethics committee, or other

institutional body to which data requests may be sent.

b) If there are no restrictions, please upload the minimal

anonymized data set necessary to replicate your study findings to a stable,

public repository and provide us with the relevant URLs, DOIs, or accession

numbers. For a list of recommended repositories, please see

https://journals.plos.org/plosone/s/recommended-repositories.

You also have the option of uploading the data as Supporting Information files,

but we would recommend depositing data directly to a data repository if

possible.

We will update your Data Availability statement on your behalf to reflect the information you provide.

4. We notice that your supplementary figures are uploaded

with the file type 'Figure'. Please amend the file type to 'Supporting Information'.

Please ensure that each Supporting Information file has a legend listed in the

manuscript after the references list.

5. We note that you have included the phrase “data not shown” in your manuscript. Unfortunately, this does not meet our data sharing requirements. PLOS does not permit references to inaccessible data. We require that authors provide all relevant data within the paper, Supporting Information files, or in an acceptable, public repository. Please add a citation to support this phrase or upload the data that corresponds with these findings to a stable

repository (such as Figshare or Dryad) and provide and URLs, DOIs, or accession

numbers that may be used to access these data. Or, if the data are not a core

part of the research being presented in your study, we ask that you remove the

phrase that refers to these data.

If the reviewer comments include a recommendation to cite specific previously published works, please review and evaluate these publications to determine whether they are relevant and should be cited. There is no requirement to cite these works unless the editor has indicated otherwise.

Additional Editor Comments (if provided):

[Note: HTML markup is below. Please do not edit.]

Reviewers' comments:

Reviewer's Responses to Questions

**Comments to the Author**

1. If the authors have adequately addressed your comments raised in a previous round of review and you feel that this manuscript is now acceptable for publication, you may indicate that here to bypass the “Comments to the Author” section, enter your conflict of interest statement in the “Confidential to Editor” section, and submit your "Accept" recommendation.

Reviewer #2: All comments have been addressed

Reviewer #3: All comments have been addressed

Reviewer #4: All comments have been addressed

**********

2. Is the manuscript technically sound, and do the data support the conclusions?

The manuscript must describe a technically sound piece of scientific research with data that supports the conclusions. Experiments must have been conducted rigorously, with appropriate controls, replication, and sample sizes. The conclusions must be drawn appropriately based on the data presented.

Reviewer #2: Yes

Reviewer #3: Yes

Reviewer #4: Partly

**********

3. Has the statistical analysis been performed appropriately and rigorously?

Reviewer #2: Yes

Reviewer #3: I Don't Know

Reviewer #4: Yes

**********

4. Have the authors made all data underlying the findings in their manuscript fully available?

The PLOS Data policy requires authors to make all data underlying the findings described in their manuscript fully available without restriction, with rare exception (please refer to the Data Availability Statement in the manuscript PDF file). The data should be provided as part of the manuscript or its supporting information, or deposited to a public repository. For example, in addition to summary statistics, the data points behind means, medians and variance measures should be available. If there are restrictions on publicly sharing data—e.g. participant privacy or use of data from a third party—those must be specified.

Reviewer #2: No

Reviewer #3: Yes

Reviewer #4: Yes

**********

5. Is the manuscript presented in an intelligible fashion and written in standard English?

PLOS ONE does not copyedit accepted manuscripts, so the language in submitted articles must be clear, correct, and unambiguous. Any typographical or grammatical errors should be corrected at revision, so please note any specific errors here.

Reviewer #2: Yes

Reviewer #3: Yes

Reviewer #4: Yes

**********

6. Review Comments to the Author

Please use the space provided to explain your answers to the questions above. You may also include additional comments for the author, including concerns about dual publication, research ethics, or publication ethics. (Please upload your review as an attachment if it exceeds 20,000 characters)

Reviewer #2: The manuscript looks in great shape now. Well done on this important and needed work. I think it will make a valuable contribution to the field.

Reviewer #3: The article is very methodically explained. However I have a few suggestions

L99-P21 the acronym P to be expanded

L165 wav" Format to be replaced by WAV

L316 "significantly prevalence" to be corrected to significant prevalence

L399 VPA treated animals displayed a bimodal distribution of mean call duration in contrast to unimodal pattern " I feel it needs some clarification of this line.

Reviewer #4: PLOS ONE PONE-D-25-43300R1

Reviewer: NN

REVIEWER COMMENTS of: Altered Stress and Fear Responses in the VPA Rat Model of Autism: Behavioral Dissociation Across Tactile, Nociceptive, and Social Contexts

The submitted material provides a comprehensive overview of stroke rehabilitation and demonstrates a strong emphasis on clinically relevant concepts, particularly the role of neuroplasticity and multidisciplinary management. However, several aspects could be improved to enhance clarity and academic quality. In terms of content, certain concepts, especially those related to neuroplasticity and the rationale behind specific rehabilitation interventions, would benefit from clearer explanation and more explicit linkage to clinical practice. Additionally, the use of terminology and formatting is somewhat inconsistent throughout the document, and standardization in accordance with established medical guidelines is recommended. The inclusion of up-to-date references would further strengthen the scientific credibility of the material, particularly for statements regarding recovery timelines and therapeutic effectiveness.

Second, some sections, particularly the description of behavioral paradigms and results, are dense and may benefit from clearer structuring.

1. Absence of hearing assessment critically undermines USV-based conclusions

The authors themselves acknowledge the lack of auditory assessment as a limitation, but this understates the problem. A significant portion of the results, particularly the emotional contagion findings, depends on the assumption that VPA and CTL observer animals perceived the demonstrator's vocalizations equally. VPA-exposed rats are known to exhibit altered auditory processing and brainstem auditory evoked potential abnormalities (see Gandal et al., 2010; Mehta & Bhatt, 2013). Without audiometric screening, it is impossible to rule out that differences in observer freezing or vocalization patterns between oV+ and oC+ animals reflect differences in auditory sensitivity rather than emotional processing. The authors must either provide hearing data or substantially qualify their emotional contagion conclusions to acknowledge this as a confound, not merely a limitation.

2. The vocalization chamber-attribution method is insufficiently validated for the main text

The power-threshold method used to assign USVs to either the demonstrator or observer chamber (>−76 dB = DEM; <−85 dB = OBS) is described only in a supplementary figure derived from a maternal separation pilot. Several issues remain unaddressed: (a) The pilot used neonatal animals at P08 and P14, whereas the emotional contagion experiment was conducted at P41: body size, lung capacity, and call amplitude change substantially across development, making cross-age threshold transfer questionable. (b) There is an unresolved gap between −76 dB and −85 dB where calls cannot be reliably attributed. What proportion of total calls fell in this ambiguous range, and how were they handled? (c) Was any cross-validation performed within the actual emotional contagion dataset? This method is central enough to the paper's main findings that it requires a more rigorous validation and should be described transparently in the Methods section, not relegated to supplementary material.

3. Litter effects are inadequately controlled

The authors argue that distributing offspring across groups constitutes sufficient litter-effect control. While this randomization strategy reduces confounding, it does not eliminate it, and the claim that statistical adjustment was therefore "unnecessary" is not well-supported. The number of litters is relatively small (8 VPA-producing litters, 6 CTL-producing litters), and some females contributed two litters. Given that litter is a recognized source of non-independence in rodent behavioral studies (Lazic & Essioux, 2013), a sensitivity analysis, such as a mixed model with litter as a random effect, or at minimum a report of ICC values, should be provided to demonstrate that litter-level clustering did not inflate false-positive rates.

4. Interpretive overreach in the fear conditioning dissociation findings

The authors present the absence of correlation between freezing, defecation, and vocalization in VPA rats as evidence of "disrupted coordination of fear responses" and "impaired integration of emotional processing." This is an interesting hypothesis, but the interpretation rests on null results from correlation analyses conducted in subgroups that are notably small (9 VPA animals for the freezing/USV correlation). The statistical power to detect correlations of moderate effect size (r ~0.5) in a sample of 9 is approximately 30–40%, meaning failure to detect a correlation is highly uninformative. The authors should either (a) calculate and report post-hoc power for these correlation analyses, or (b) substantially temper their interpretive language to reflect that absence of evidence is not evidence of disrupted integration, particularly in underpowered subgroup analyses.

5. Inconsistent block-level analysis across paradigms

The fear conditioning and emotional contagion paradigms use block-by-block analysis (B0–B5), but the criteria for what constitutes a "responsive" animal differ across measures and are not consistently applied. For freezing, responsiveness is defined as displaying any freezing during the experiment; for vocalizations, it is defined as emitting at least one USV. These thresholds are reasonable individually, but the resulting subgroup sizes shift considerably across analyses within the same experiment (e.g., N = 27 for freezing vs. N = 22 for USV in CTL fear conditioning).

Overall, the content is informative and relevant, and with refinement in structure, clarity, and evidence support, it has strong potential to serve as an effective educational resource.

**********

7. PLOS authors have the option to publish the peer review history of their article (what does this mean?). If published, this will include your full peer review and any attached files.

If you choose “no”, your identity will remain anonymous but your review may still be made public.

**Do you want your identity to be public for this peer review?**  For information about this choice, including consent withdrawal, please see our Privacy Policy.

Reviewer #2: No

Reviewer #3: **Yes:**  Shabina Ahmed

Reviewer #4: No

**********

[NOTE: If reviewer comments were submitted as an attachment file, they will be attached to this email and accessible via the submission site. Please log into your account, locate the manuscript record, and check for the action link "View Attachments". If this link does not appear, there are no attachment files.]

To ensure your figures meet our technical requirements, please review our figure guidelines: https://journals.plos.org/plosone/s/figures

You may also use PLOS’s free figure tool, NAAS, to help you prepare publication quality figures: https://journals.plos.org/plosone/s/figures#loc-tools-for-figure-preparation.

NAAS will assess whether your figures meet our technical requirements by comparing each figure against our figure specifications.

# Author comment: Author response to Decision Letter 2

**DOI:** 10.1371/journal.pone.0353839.r004

8 Jun 2026

Answers to the Reviewers’ Comments

Dear Dr. Saka - Academic Editor and the Editorial Board of PLOS ONE,

Thank you for the opportunity to submit our revised manuscript, titled "Altered Stress and Fear Responses in the VPA Rat Model of Autism: Behavioral Dissociation Across Tactile, Nociceptive, and Social Contexts." We would like to express our sincere gratitude to the reviewers for their time, dedication, and highly constructive feedback during this evaluation process. We believe the quality and clarity of the paper have significantly improved with the revisions as we have meticulously considered and addressed each of the commentaries and suggestions. Below, we provide a comprehensive, point-by-point response detailing the modifications implemented, which we believe have significantly strengthened the final manuscript.

Reviewer #2: The manuscript looks in great shape now. Well done on this important and needed work. I think it will make a valuable contribution to the field.

R. We thank the reviewer for the comments.

Reviewer #3: The article is very methodically explained. However I have a few suggestions

L99-P21 the acronym P to be expanded

R: We thank the reviewer for the comment. We clarified the acronym in the Manuscript as follows “... postnatal day 21 (i.e. P21).” In L100.

L165 wav" Format to be replaced by WAV~

R: We thank the reviewer for the comment. We changed it to “WAV” in all places it appeared in the Manuscript. In L170.

L316 "significantly prevalence" to be corrected to significant prevalence

R: R: We thank the reviewer for the comment. We revised the Manuscript text.

Originally: “During the electro-tactile sensitivity test (Fig 2A), the VPA group showed a significant prevalence of animals releasing fecal pellets as compared to the CTL group (Fig 2B).”)

Now reads: “During the electro-tactile sensitivity test (Fig 2A), a larger proportion of VPA-treated rats released fecal pellets compared with CTL rats (Fig 2B), indicating increased stress susceptibility.”In L321.

L399 VPA treated animals displayed a bimodal distribution of mean call duration in contrast to unimodal pattern " I feel it needs some clarification of this line.

R: We thank the reviewer for the comment. We clarified this point in the Manuscript by adding the following explanation:

“ vocalizations emitted by VPA-treated rats displayed a bimodal distribution of call duration, characterized by clusters of shorter (~500 ms) and longer (~800 ms) calls, whereas CTL rats exhibited a more unimodal distribution centered around ~600 ms.” L405.

Reviewer #4: PLOS ONE PONE-D-25-43300R1

Reviewer: NN

REVIEWER COMMENTS of: Altered Stress and Fear Responses in the VPA Rat Model of Autism: Behavioral Dissociation Across Tactile, Nociceptive, and Social Contexts

The submitted material provides a comprehensive overview of stroke rehabilitation and demonstrates a strong emphasis on clinically relevant concepts, particularly the role of neuroplasticity and multidisciplinary management. However, several aspects could be improved to enhance clarity and academic quality. In terms of content, certain concepts, especially those related to neuroplasticity and the rationale behind specific rehabilitation interventions, would benefit from clearer explanation and more explicit linkage to clinical practice. Additionally, the use of terminology and formatting is somewhat inconsistent throughout the document, and standardization in accordance with established medical guidelines is recommended. The inclusion of up-to-date references would further strengthen the scientific credibility of the material, particularly for statements regarding recovery timelines and therapeutic effectiveness.

Second, some sections, particularly the description of behavioral paradigms and results, are dense and may benefit from clearer structuring.

R: The opening comments of the reviewer are not related to our Manuscript. Our Manuscript has not investigated “... stroke rehabilitation…”.

1. Absence of hearing assessment critically undermines USV-based conclusions

The authors themselves acknowledge the lack of auditory assessment as a limitation, but this understates the problem. A significant portion of the results, particularly the emotional contagion findings, depends on the assumption that VPA and CTL observer animals perceived the demonstrator's vocalizations equally. VPA-exposed rats are known to exhibit altered auditory processing and brainstem auditory evoked potential abnormalities (see Gandal et al., 2010; Mehta & Bhatt, 2013). Without audiometric screening, it is impossible to rule out that differences in observer freezing or vocalization patterns between oV+ and oC+ animals reflect differences in auditory sensitivity rather than emotional processing. The authors must either provide hearing data or substantially qualify their emotional contagion conclusions to acknowledge this as a confound, not merely a limitation.

R: We thank the reviewer for this important observation. We agree that auditory function represents a potential confounding factor when interpreting behaviors dependent on acoustic communication, particularly in the emotional contagion paradigm. Direct audiometric or auditory brainstem response measurements were not performed in the present study, and therefore we cannot fully exclude the possibility that subtle alterations in auditory processing contributed to the observed behavioral differences between VPA-treated and control animals. Accordingly, we have revised the Discussion to more explicitly acknowledge this point as a potential confound when interpreting emotional contagion-related behaviors (L710).

Previous electrophysiological work from our group (Anomal et al., 2015) suggests that cortical responsiveness to ultrasonic-frequency stimuli remains largely preserved in the VPA rat model. Using high-density recordings in primary auditory cortex (A1), we observed that 36% of recording sites in VPA-treated rats responded to high-frequency (10–50 kHz), low-intensity (<50 dB) stimuli, compared to 40% in control animals, indicating only modest differences in cortical responsiveness to ultrasonic sounds. We therefore interpret our findings cautiously: while existing evidence suggests that gross sensitivity to high-frequency auditory stimuli is relatively preserved in VPA-treated rats, we cannot exclude the possibility that more subtle alterations in auditory perception, salience attribution, or acoustic processing contributed to the behavioral effects observed in the emotional contagion paradigm.

Regarding the study by Daniel Gandal, we note that the experiments were conducted in mice rather than rats, and used low-frequency auditory brainstem response paradigms (6–9 kHz tones), which differ substantially from the ultrasonic distress vocalizations analyzed in the present study. While these findings support the possibility of altered auditory processing in VPA models, species and stimulus differences limit direct extrapolation to the present experimental context. Concerning the citation to Mehta & Bhatt (2013), we were unable to identify a publication by these authors directly related to auditory processing or emotional contagion in the VPA model.

2. The vocalization chamber-attribution method is insufficiently validated for the main text

The power-threshold method used to assign USVs to either the demonstrator or observer chamber (>−76 dB = DEM; <−85 dB = OBS) is described only in a supplementary figure derived from a maternal separation pilot. Several issues remain unaddressed: (a) The pilot used neonatal animals at P08 and P14, whereas the emotional contagion experiment was conducted at P41: body size, lung capacity, and call amplitude change substantially across development, making cross-age threshold transfer questionable. (b) There is an unresolved gap between −76 dB and −85 dB where calls cannot be reliably attributed. What proportion of total calls fell in this ambiguous range, and how were they handled? (c) Was any cross-validation performed within the actual emotional contagion dataset? This method is central enough to the paper's main findings that it requires a more rigorous validation and should be described transparently in the Methods section, not relegated to supplementary material.

R: We thank the reviewer for this important methodological observation. Because chamber attribution of ultrasonic vocalizations is central to the interpretation of the emotional contagion experiments, we agree that the procedure required clearer justification and more transparent description in the main text. To address this concern, we revised the Methods section (paragraph starting at L208) to include: (1) the rationale underlying the power-threshold attribution approach; (2) the empirical basis used to define the intensity cut-offs; and (3) the handling of calls falling within the ambiguous intensity range. Supplementary Figure S1 was also simplified and now serves as supporting validation material rather than the primary methodological description.

Regarding developmental differences in call amplitude (comment a), we agree that body size and lung maturation influence USV intensity across development. To address this issue, Figure S1 includes recordings not only from neonatal maternal separation paradigms (P08 and P14), but also from juvenile fear conditioning experiments (P35–38), which more closely approximate the age and emotional context of the emotional contagion experiments (P41). Across all conditions, calls recorded from the same chamber as the emitter consistently exhibited substantially higher intensities than calls recorded from the opposite chamber, supporting the use of conservative intensity thresholds for chamber attribution. Importantly, the thresholds were intentionally selected to minimize misclassification rather than maximize call inclusion. Vocalizations above −76 dB were classified as demonstrator-derived, whereas vocalizations below −85 dB were classified as observer-derived. Calls within the intermediate range (−76 to −85 dB), which could not be reliably assigned to either chamber, were excluded from analysis.

Regarding the ambiguous range (comment b), calls between −76 and −85 dB represented 9.8% of all vocalizations recorded during the emotional contagion experiments (2086/21388 calls; CTL: 8.4%; VPA: 11.2%). Consequently, 91.2% of all calls remained available for analysis after exclusion of potentially ambiguous signals. This exclusion procedure was adopted specifically to reduce chamber-attribution uncertainty. Regarding cross-validation within the emotional contagion dataset (comment c), we acknowledge that no direct ground-truth validation of emitter identity was performed during these sessions. We now explicitly recognize this as a methodological limitation in the revised manuscript (L710). Nevertheless, the use of conservative cut-offs together with exclusion of intermediate-intensity calls was intended to minimize assignment ambiguity and reduce the likelihood of systematic misclassification.

Finally, all methodological details regarding chamber attribution thresholds and exclusion criteria have now been incorporated into the main Methods section (paragraph starting at L208), rather than being described exclusively in the Supplementary Materials.

Included in the Discussion section (L660):

“Although we cannot entirely exclude the possibility that subtle differences in auditory sensitivity contribute to the observed behavioral variations between VPA-treated and control animals, our previous research indicates a difference of less than 5% in cortical sensitivity for high-frequency sounds. Specifically, high-density cortical recordings showed that 36% of A1 sites in VPA-treated rats were responsive to high-frequency (10–50 kHz), low-intensity (<50 dB) stimuli, compared to 40% in control animals (68)”.

3. Litter effects are inadequately controlled

The authors argue that distributing offspring across groups constitutes sufficient litter-effect control. While this randomization strategy reduces confounding, it does not eliminate it, and the claim that statistical adjustment was therefore "unnecessary" is not well-supported. The number of litters is relatively small (8 VPA-producing litters, 6 CTL-producing litters), and some females contributed two litters. Given that litter is a recognized source of non-independence in rodent behavioral studies (Lazic & Essioux, 2013), a sensitivity analysis, such as a mixed model with litter as a random effect, or at minimum a report of ICC values, should be provided to demonstrate that litter-level clustering did not inflate false-positive rates.

We thank the reviewer for raising this important point. We agree that balanced allocation of offspring across experimental groups reduces, but does not eliminate, potential litter-related non-independence. Accordingly, in the revised analyses we explicitly evaluated litter-level clustering using linear mixed-effects models including litter as a random effect, consistent with the recommendations of Lazic & Essioux.

As part of the experimental design, offspring from multiple litters were distributed across experimental conditions to minimize potential confounding between treatment and litter identity. In each experiment, independent litters were used for the CTL and VPA groups, such that no litter contributed animals to both treatment conditions within the same experimental paradigm. Detailed litter composition for each experiment has now been included in the Supplementary Materials (Table S1). Overall, the study included animals derived from 14 independent litters (6 CTL-derived and 8 VPA-derived litters). Some litters contributed offspring to more than one behavioral experiment.

Table S1: Summary table of all animals and litters used in the experiments. Offspring from multiple litters were distributed across experimental conditions to minimize potential confounding between treatment and litter identity. In each experiment, independent litters were used for the CTL and VPA groups, such that no litter contributed animals to both treatment conditions within the same experimental paradigm.

Experiment

Group

Litters

Dam-Litter ID

N/Litter

Touch sensitivity & Electro-tactile sensitivity experiments

CTL

5

(D03L1, D05L1, D09L1, D13L1, D17L1)

[13, 8, 3, 3, 3]

VPA

5

(D02L2, D04L1, D10L2, D15L1, D18L2)

[12, 8, 1, 4, 3]

Nociceptive stress response & Fear Conditioning experiments

CTL

4

(D07L1, D09L1, D13L1, D17L1)

[8, 8 ,8, 7]

VPA

5

(D03L2, D10L2, D11L1, D15L1, D16L2)

[8, 8, 3, 8, 3]

Emotional Contagion experiment

oC-

4

(D07L1, D09L1, D13L1, D17L1)

[4, 4, 4, 3]

oC+

4

(D07L1, D09L1, D13L1, D17L1)

[4, 4, 4, 4]

oV-

5

(D03L2, D10L2, D11L1, D15L1, D16L2)

[4, 4, 1, 3, 3]

oV+

4

(D03L2, D10L2, D11L1, D15L1)

[4, 4, 2, 5]

To directly address the reviewer’s concern, we performed complementary mixed-effects sensitivity analyses on the emotional contagion dataset.

First, we analyzed the individual-animal repeated-measures data using a linear mixed-effects model taking into account litter as random effect. The selected model included Group, Treatment, and Treatment × Block as fixed effects, with random intercepts for litter and individual animals nested within litter. This analysis revealed a highly significant Treatment × Block interaction (F(5,352) = 10.729, p = 1.30 × 10−9), indicating that freezing dynamics across experimental blocks differed as a function of treatment condition. Importantly, the estimated litter intraclass correlation coefficient was extremely small: ICC_Litter = 0.0069 indicating that less than 1% of the total variance was attributable to litter of origin, i.e., animals from the same litter behaved almost independently with respect to the analyzed outcome. In contrast, repeated measures within individual animals accounted for a substantially larger proportion of variance, supporting the conclusion that the observed behavioral effects primarily reflected individual behavioral dynamics rather than litter-specific clustering. Second, as a conservative sensitivity analysis, we averaged freezing values within litters and reanalyzed the data using litter-level observations. Despite the substantial reduction in statistical

Attachment Submitted filename: Response to Reviewers.docx

# Peer review: Decision Letter 2

**DOI:** 10.1371/journal.pone.0353839.r005

**Authors:** Hualin Fu, Vara Prasad Saka, Vara Prasad Saka

30 Jun 2026

<p>Altered Stress and Fear Responses in the VPA Rat Model of Autism: Behavioral Dissociation Across Tactile, Nociceptive, and Social Contexts

PONE-D-25-43300R2

Dear Dr. Hashiguchi,

We’re pleased to inform you that your manuscript has been judged scientifically suitable for publication and will be formally accepted for publication once it meets all outstanding technical requirements.

Within one week, you’ll receive an e-mail detailing the required amendments. When these have been addressed, you’ll receive a formal acceptance letter and your manuscript will be scheduled for publication.

An invoice will be generated when your article is formally accepted. Please note, if your institution has a publishing partnership with PLOS and your article meets the relevant criteria, all or part of your publication costs will be covered. Please make sure your user information is up-to-date by logging into Editorial Manager at Editorial Manager® and clicking the ‘Update My Information' link at the top of the page. For questions related to billing, please contact billing support.

If your institution or institutions have a press office, please notify them about your upcoming paper to help maximize its impact. If they’ll be preparing press materials, please inform our press team as soon as possible -- no later than 48 hours after receiving the formal acceptance. Your manuscript will remain under strict press embargo until 2 pm Eastern Time on the date of publication. For more information, please contact onepress@plos.org.

Kind regards,

Vara Prasad Saka

Academic Editor

PLOS One

Additional Editor Comments (optional):

Reviewers' comments:

Reviewer's Responses to Questions

**Comments to the Author**

1. If the authors have adequately addressed your comments raised in a previous round of review and you feel that this manuscript is now acceptable for publication, you may indicate that here to bypass the “Comments to the Author” section, enter your conflict of interest statement in the “Confidential to Editor” section, and submit your "Accept" recommendation.

Reviewer #2: All comments have been addressed

Reviewer #3: All comments have been addressed

**********

2. Is the manuscript technically sound, and do the data support the conclusions?

The manuscript must describe a technically sound piece of scientific research with data that supports the conclusions. Experiments must have been conducted rigorously, with appropriate controls, replication, and sample sizes. The conclusions must be drawn appropriately based on the data presented.

Reviewer #2: Yes

Reviewer #3: Yes

**********

3. Has the statistical analysis been performed appropriately and rigorously?

Reviewer #2: Yes

Reviewer #3: I Don't Know

**********

4. Have the authors made all data underlying the findings in their manuscript fully available?

The PLOS Data policy requires authors to make all data underlying the findings described in their manuscript fully available without restriction, with rare exception (please refer to the Data Availability Statement in the manuscript PDF file). The data should be provided as part of the manuscript or its supporting information, or deposited to a public repository. For example, in addition to summary statistics, the data points behind means, medians and variance measures should be available. If there are restrictions on publicly sharing data—e.g. participant privacy or use of data from a third party—those must be specified.

Reviewer #2: Yes

Reviewer #3: Yes

**********

5. Is the manuscript presented in an intelligible fashion and written in standard English?

PLOS ONE does not copyedit accepted manuscripts, so the language in submitted articles must be clear, correct, and unambiguous. Any typographical or grammatical errors should be corrected at revision, so please note any specific errors here.

Reviewer #2: Yes

Reviewer #3: Yes

**********

6. Review Comments to the Author

Please use the space provided to explain your answers to the questions above. You may also include additional comments for the author, including concerns about dual publication, research ethics, or publication ethics. (Please upload your review as an attachment if it exceeds 20,000 characters)

Reviewer #2: All my comments were already addressed in the previous revision. I reviewed the new changes in response to the other reviewers, and the manuscript continues to be in a good shape for publication. I have no further concerns. Thank you.

Reviewer #3: The authors have adequately addressed reviewers comments and the manuscript is technically sound. However I have a suggestion that expanded terms of the acronyms to be included eg L193 while served as B0, L648 ASD and TD peers,

**********

7. PLOS authors have the option to publish the peer review history of their article (what does this mean?). If published, this will include your full peer review and any attached files.

If you choose “no”, your identity will remain anonymous but your review may still be made public.

**Do you want your identity to be public for this peer review?**  For information about this choice, including consent withdrawal, please see our Privacy Policy.

Reviewer #2: No

Reviewer #3: **Yes:**  Shabina Ahmed

**********

# Editor report: Acceptance letter

**DOI:** 10.1371/journal.pone.0353839.r006

**Authors:** Hualin Fu, Vara Prasad Saka, Vara Prasad Saka

PONE-D-25-43300R2

PLOS One

Dear Dr. Hashiguchi,

I'm pleased to inform you that your manuscript has been deemed suitable for publication in PLOS One. Congratulations! Your manuscript is now being handed over to our production team.

At this stage, our production department will prepare your paper for publication. This includes ensuring the following:

* All references, tables, and figures are properly cited

* All relevant supporting information is included in the manuscript submission,

* There are no issues that prevent the paper from being properly typeset

You will receive further instructions from the production team, including instructions on how to review your proof when it is ready. Please keep in mind that we are working through a large volume of accepted articles, so please give us a few days to review your paper and let you know the next and final steps.

Lastly, if your institution or institutions have a press office, please let them know about your upcoming paper now to help maximize its impact. If they'll be preparing press materials, please inform our press team within the next 48 hours. Your manuscript will remain under strict press embargo until 2 pm Eastern Time on the date of publication. For more information, please contact onepress@plos.org.

You will receive an invoice from PLOS for your publication fee after your manuscript has reached the completed accept phase. If you receive an email requesting payment before acceptance or for any other service, this may be a phishing scheme. Learn how to identify phishing emails and protect your accounts at https://explore.plos.org/phishing.

If we can help with anything else, please email us at customercare@plos.org.

Thank you for submitting your work to PLOS ONE and supporting open access.

Kind regards,

PLOS ONE Editorial Office Staff

on behalf of

Dr. Vara Prasad Saka

Academic Editor

PLOS One
